# Distinct patterns of auto-reactive antibodies associated with organ-specific immune-related adverse events

**DOI:** 10.3389/fimmu.2023.1322818

**Published:** 2023-12-12

**Authors:** Mehmet Altan, Quan-Zhen Li, Qi Wang, Natalie I. Vokes, Ajay Sheshadri, Jianjun Gao, Chengsong Zhu, Hai T. Tran, Saumil Gandhi, Mara B. Antonoff, Stephen Swisher, Jing Wang, Lauren A. Byers, Noha Abdel-Wahab, Maria C. Franco-Vega, Yinghong Wang, J. Jack Lee, Jianjun Zhang, John V. Heymach

**Affiliations:** ^1^ Department of Thoracic/Head and Neck Medical Oncology, The University of Texas MD Anderson Cancer Center, Houston, TX, United States; ^2^ Department of Immunology, UT Southwestern Medical Center, Dallas, TX, United States; ^3^ Department of Biostatistics, The University of Texas MD Anderson Cancer Center, Houston, TX, United States; ^4^ Department of Pulmonary Medicine, The University of Texas MD Anderson Cancer Center, Houston, TX, United States; ^5^ Department of Genitourinary Medical Oncology, The University of Texas MD Anderson Cancer Center, Houston, TX, United States; ^6^ Department of Radiation Oncology, The University of Texas MD Anderson Cancer Center, Houston, TX, United States; ^7^ Department of Thoracic and Cardiovascular Surgery, The University of Texas MD Anderson Cancer Center, Houston, TX, United States; ^8^ Department of General Internal Medicine, The University of Texas MD Anderson Cancer Center, Houston, TX, United States; ^9^ Department of Hospital Medicine, The University of Texas MD Anderson Cancer Center, Houston, TX, United States; ^10^ Department of Gastroenterology Hepatology and Nutrition, The University of Texas MD Anderson Cancer Center, Houston, TX, United States

**Keywords:** NSCLC, auto-reactive antibodies, immune related adverse events, immune checkpoint inhibitor, pneumonitis

## Abstract

The roles of preexisting auto-reactive antibodies in immune-related adverse events (irAEs) associated with immune checkpoint inhibitor therapy are not well defined. Here, we analyzed plasma samples longitudinally collected at predefined time points and at the time of irAEs from 58 patients with immunotherapy naïve metastatic non-small cell lung cancer treated on clinical protocol with ipilimumab and nivolumab. We used a proteomic microarray system capable of assaying antibody reactivity for IgG and IgM fractions against 120 antigens for systemically evaluating the correlations between auto-reactive antibodies and certain organ-specific irAEs. We found that distinct patterns of auto-reactive antibodies at baseline were associated with the subsequent development of organ-specific irAEs. Notably, ACHRG IgM was associated with pneumonitis, anti-cytokeratin 19 IgM with dermatitis, and anti-thyroglobulin IgG with hepatitis. These antibodies merit further investigation as potential biomarkers for identifying high-risk populations for irAEs and/or monitoring irAEs during immunotherapy treatment.

**Trial registration:**

ClinicalTrials.gov identifier: NCT03391869.

## 1 Introduction

Immune checkpoint inhibitors (ICIs), such as cytotoxic T-lymphocyte–associated protein 4 (CTLA-4) and programmed cell death protein-1 (PD-1)/programmed cell death ligand-1 (PD-L1) inhibitors, produce durable clinical responses in various solid tumors, including non-small cell lung cancer (1, 2). The immune toxicity of ICIs, termed immune-related AEs (irAEs), result from organ inflammation outside of the cancer. In contrast to the well-characterized temporal patterns of toxicities arising from chemotherapy or targeted therapy, the onset and duration of irAEs are unpredictable, and predisposing factors for the development of irAEs not well defined (3).

Immune self-tolerance in humans is partly maintained by the inhibition of auto-reactive T cells through CTLA-4 and the PD-1/PD-L1 pathway (4, 5), and PD-1 and CTLA-4 polymorphisms are associated with various autoimmune conditions (6–8). Therefore, it is not surprising that irAEs of ICIs share clinical features with autoimmune conditions. Current evidence suggests that irAEs occur through a variety of mechanisms that involve cellular and humoral immunity, including the disruption of hemostasis by the peripheral negative selection of lymphocytes with anti-CTLA-4 therapy, which promotes the expansion of self-reactive T cells; alteration of the epigenome of exhausted T cells by inhibition of the PD-1/PD-L1 pathway; and hampering peripheral tolerance by the depletion of regulatory cells, molecular mimicry, epitope spread, and auto-reactive antibodies (8–12).

Previous studies show that approximately 8-9% of the US population has an autoimmune disease and that a quarter of healthy individuals have strong IgG humoral responses to a variety of self-antigens that may be relevant to irAEs (13, 14). Seropositivity in patients with irAEs has been demonstrated in case reports and observational cohorts (8, 15, 16). However, conclusions to date have been limited by cohort size, lack of longitudinal sample collection, and heterogeneity of ICI treatments. A previous work shed light on the impact of auto-reactive antibodies that exist prior to ICI therapy on the risk for developing irAEs, including hypophysitis and pneumonitis (8). In this study, we systematically analyzed a larger set of longitudinally collected patient plasma samples to identify pre-existing auto-reactive antibodies, determine their temporal dynamics with ICI treatment, and correlate them with the development of a wider spectrum of irAEs.

## 2 Patients and methods

### 2.1 Clinical data and sample collection

Longitudinal patient plasma samples were collected from the ongoing LONESTAR clinical study (ClinicalTrials.gov identifier: NCT03391869) conducted at the University of Texas MD Anderson Cancer. This open-label, single-center, randomized clinical study enrolled patients with histologically or cytologically confirmed metastatic NSCLC. Key exclusion criteria included prior immunotherapy or more than one prior line of chemotherapy, tumors harboring EGFR-sensitizing mutations or ALK fusions eligible for standard-of-care targeted therapies, and active, known, or suspected autoimmune disease. The protocol and all amendments were approved by the Institutional Review Board (#2017-0311). All patients provided written informed consent to participate in the study, including blood collection for auto-reactive antibody profile analysis. In the parent trial, eligible patients received ipilimumab 1 mg/kg every six weeks and nivolumab 3mg/kg every two weeks (I+N) for 12 weeks (induction); those patients who did not experience disease progression were then randomly assigned to local consolidative therapy (LCT) with radiation and surgery for residual disease vs. no LCT. A synopsis of the study protocol is available in the **Supplementary File**, and the study schema is provided in **Supplementary Figure 1**.

**Figure 1:**
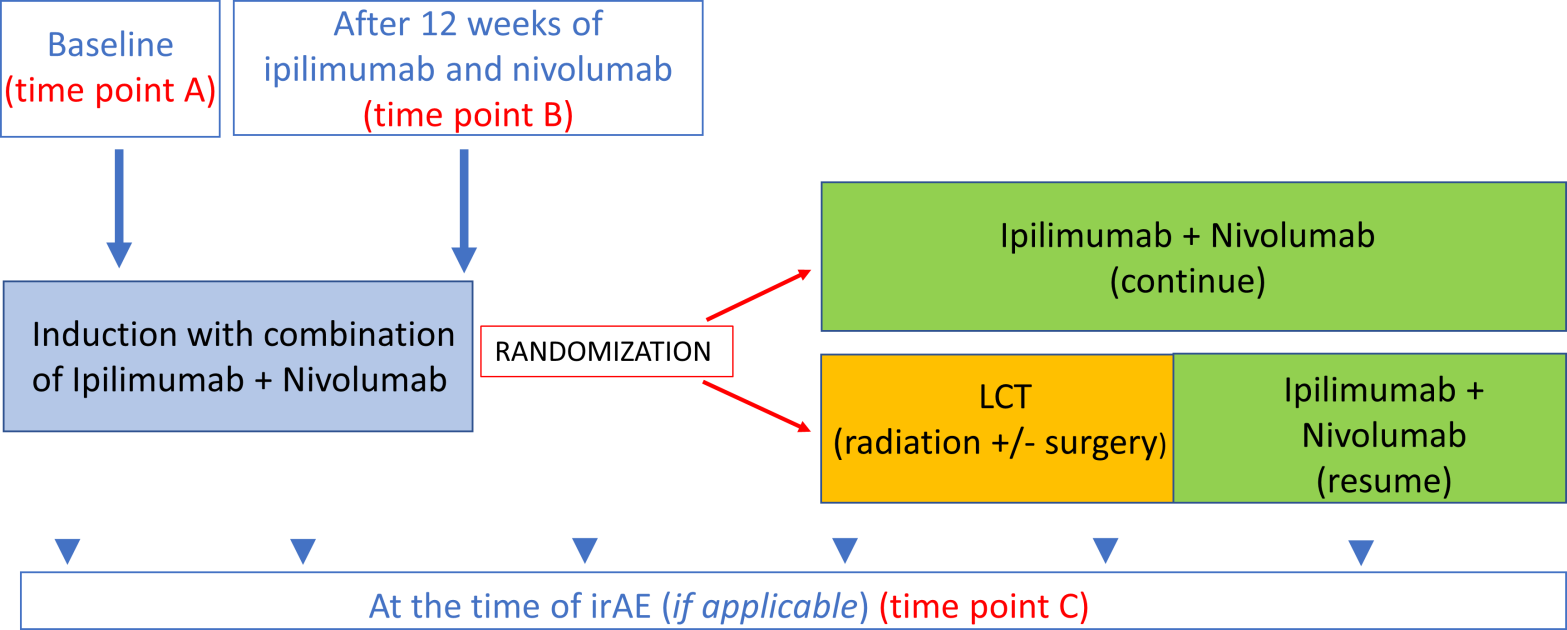
Time points for blood collection. Time point A baseline (prior to ICI therapy). Time point B After 12 weeks of ICI therapy 12-week samples (this time point also used as control for treated patients with no irAEs. Time point C At the time of irAE (if applicable).

Plasma samples were collected at 1) baseline (prior to I+N therapy, on the same day as cycle 1 of therapy) (time point A), 2) after I+N induction (12 weeks after cycle 1) (time point B), and 3) at the time of grade ≥ 2 irAEs in patients who developed toxicities (time point C) (**Figure 1**). IrAEs were prospectively collected and graded using the Common Terminology Criteria for Adverse Events v5.0. IrAE samples were obtained within 14 days of symptom onset during the clinic visit. The attribution of adverse events were made by available clinical, laboratory, and histologic data and reported per the NCI adverse reporting guidelines (17). In this study, only four organ-specific irAEs were included in the analysis due to availability of number of samples (pneumonitis, dermatitis, diarrhea/colitis, and hepatitis). Samples from patients who were exposed to 12-weeks of ICI induction therapy and did not experience any irAEs within at least 6 months of follow-up were used as “controls” for comparative analyses. Patients with overlapping toxicities (simultaneous irAEs > 1) were excluded from this analysis.

We analyzed the IgG and IgM auto-reactive antibody panel for each irAE (pneumonitis, dermatitis, hepatitis, colitis) and 1) compared auto-reactive antibody panels at baseline in patients who had organ-specific irAEs vs. no irAE (time point A for irAE vs. no irAE), 2) compared auto-reactive antibody panels at the time of irAE onset with 12-week samples from treated patients with no irAEs (control) (time point B vs. time point C), and 3) analyzed the longitudinal auto-reactive antibody changes in patients with an organ-specific toxicity by comparing their baseline sample with toxicity samples (time point A vs. C) (**Figure 1**).

### 2.2 Microarray protein profiling

Human serum samples from all subjects were collected, aliquoted, and stored at -80°C. Autoantigen microarrays were manufactured at the Microarray Core Facility of The University of Texas Southwestern Medical Center (Dallas, Texas, USA). One hundred twenty autoantigens, including nuclear antigens, cytosolic antigens, and tissue-specific antigens, were selected from previously known autoantibodies in various immune-related diseases (e.g., cancer and allergic disease) (18) on the basis of the published literature (a full list of the auto-reactive antibodies used in this panel is shown in **Supplementary Table 1**) and as previously described (19, 20). Four internal control proteins (human IgG, human IgM, anti–human IgG, and anti-human IgM), each at four different concentrations (0.1 mg/mL, 0.05 mg/mL, 0.025, and 0.0125 mg/mL), were imprinted on the arrays as positive and normalization controls.

Serum samples were pretreated with DNAse-I and diluted 1:50 in PBST buffer for autoantibody profiling. The samples were incubated with autoantigen arrays, and autoantibodies bound to arrayed proteins were measured with cy3-conjugated anti-human IgG (1:1000, Jackson ImmunoResearch, West Grove, Pennsylvania, USA) and cy5-conjugated anti-human IgM (1:2000, Jackson ImmunoResearch) using a Genepix 4400A scanner (Molecular Devices, Sunnyvale, California, USA) with laser wavelengths of 532 nm and 635 nm. The resulting images were analyzed using Genepix Pro 7.0 software (Molecular Devices).

The median signal intensity for each spot was calculated and subtracted from the local background around the spot, and the data obtained from the duplicate spots were averaged. The background-subtracted signal intensity of each antigen was normalized to the average intensity of human IgG or IgM, which were spotted on the array as an internal control. Finally, the net fluorescence intensity (NFI) was generated as a quantitative measurement of the binding capacity of each antibody with the corresponding autoantigen, normalized with a robust linear model using a built-in Ig control with various dilutions (21). A signal-to-noise ratio (SNR) was generated for each antigen. The SNR was used as a quantitative measure of the ability to resolve the true signal from background noise. To avoid outliers in the NFI or SNR, the autoantibody score, defined by log2((NFI × SNR) + 1), was used for all downstream analyses. Autoantibody scores were used in the downstream analysis.

### 2.3 Statistical analysis

A paired t-test was used to compare the means of longitudinally collected baseline and toxicity samples. Post-induction (minimum 12-week exposure to combination ICI therapy) samples from patients who had no irAEs during the study period were used as a control compared with the toxicity samples. An unpaired t-test was used to compare differences between groups. The resulting p-values were adjusted using the Benjamini and Hochberg method to control for the type I error rate of multiple comparisons (22). P values were two-tailed for all analyses, *p* ≤ 0.05 was considered statistically significant. *P* values and corresponding false discovery rates (FDR) were defined for each marker and are provided in the **Supplementary File**.

To remove the batch effect, a modified mean centering batch correction was applied, where the baseline samples in each batch served as control samples during the mean centering batch correction process (**Supplementary Table 2**) (23).

## 3 Results

### 3.1 Baseline characteristics

One hundred and eighty-nine patients were enrolled in the trial at the time of data lock in January 2021, and samples from 58 patients who had prospective follow-up for a minimum of 6 months and had more than one timepoint of blood collection were included in the analysis. All patients had a baseline sample (pretreatment, immunotherapy-naïve), and 54 patients had a sample at 12-weeks of ipilimumab and nivolumab therapy (post induction); 43 grade ≥ 2 irAE events from 41 patients had corresponding blood collection:10 patients with pneumonitis, 12 with dermatitis, 15 with diarrhea/colitis, and 6 with hepatitis. Two patients had more than one grade ≥ 2 organ-specific toxicity (separate time points). The organ-specific involvement, grading, and timing of these toxicities are included in **Supplementary Table 3**. Baseline and post induction serum samples from 17 patients who received at least 12 weeks of ipilimumab and nivolumab and had no irAEs within 6 months of follow-up were used as “control” samples.

The final analysis included 10 pneumonitis events, seven were grade 2 and three were grade 3. Twelve grade 2 irAEs were grouped dermatitis, which included eczematous, morbilliform, acneiform, and psoriasiform rashes, pruritis, and vitiligo. Fifteen diarrhea/colitis irAEs were captured, including eight grade 2 and seven grade 3 irAEs. The events grouped in the hepatitis analysis included samples from patients with transaminitis with and without changes in liver synthetic capacity. There were six events in this category, including four grade 2 and two grade 3.

### 3.2 Auto reactive antibodies at baseline

Baseline samples from patients with pneumonitis, dermatitis, diarrhea/colitis, and hepatitis were compared with baseline samples from patients who did not experience irAEs. The full list of auto-reactive antibodies that showed higher titers in toxicity groups at baseline is provided in the **Supplementary Table 4-6**, whereas the top three statistically most significantly increased auto-reactive antibodies for IgG and IgM fractions for each organ-specific toxicity of interest (p ≤0.05), and their corresponding p and FDR values are provided in **Table 1**.

**Table 1:** Top 3 auto reactive antibodies for Ig G and Ig M fractions for each organ specific toxicity of interest with statistical significance, and their corresponding p and FDR values provided in table 1 (p ≤ 0.05 used for statistical cutoff).

	Ig G	P value	FDR	Ig M	P value	FDR
**Pneumonitis**	Cytokeratin 19 Ag	0.004843	0.619958	OmpC (E coli outer membrane porin)	0.00047	0.057215
	IL-17A	0.019349	0.98776	HSV-1 and HSV-2 Ag	0.002661	0.057215
	CA242	0.034922	0.98776	IL-2	0.002973	0.057215
**Dermatitis**	dsDNA	0.038594	0.997145	IFN alpha and beta	0.018835	0.456247
				IL-2	0.024212	0.456247
				Calreticulin	0.041953	0.456247
**Diarrhea/Colitis**	None			Hepatitis A antigen	0.008004	0.261828
				Thrombopoietin	0.008191	0.261828
				VEGF-165	0.012916	0.261828
**Hepatitis**	None			CDK2	0.007867	0.351345
				IL-2	0.017962	0.351345
				Troponin I-T-C ternary complex mixture	0.018433	0.351345

### 3.3 Longitudinal changes at toxicity: pneumonitis

**Figure 2:**
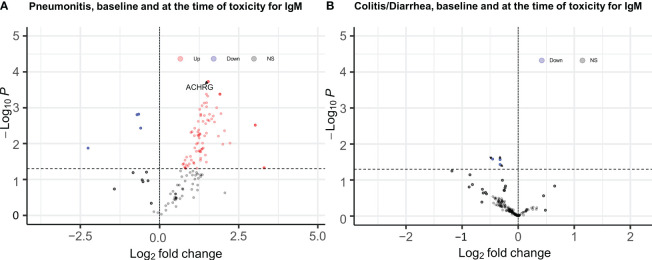
EnhancedVolcano plots for longitudinal analysis of baseline and time of toxicity auto reactive antibodies **(A)** IgM antibodies for pneumonitis, **(B)** IgM antibodies for Diarrhea/colitis, Dashed line at y axis is representing p ≤ 0.05 cut off, red color coding is representing increased autoreactive antibodies after a log transformation that are statistically significany per p value cut-off (ACHRG highlighted in **A**).

When samples obtained at the time of pneumonitis were compared with 12-week samples from controls, we observed higher titers of multiple IgG and IgM auto-reactive antibodies, with the strongest associations observed for IgG antibodies to CA125 (p= 0.001; FDR=0.03), CMV EXT (P= 0.01, FDR=0.13), and Angiotensin II type 1 receptor (p=0.01, FDR=0.16), and IgM fraction for ACHRG (p=0.00018, FDR=0.12), NSE (p=0.004, FDR=0.12), and BAFF (p=0.004, FDR=0.12) **Figure 2A** (full list provided in **Supplementary Tables 4**, **5**).

**Figure 3:**
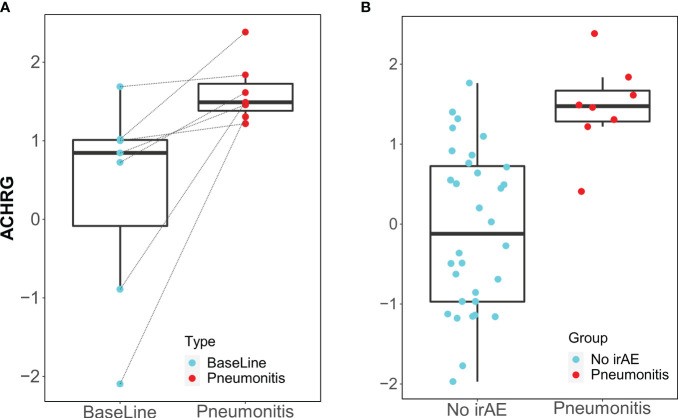
Box plots of IgM antibody for ACHRG. **(A)** Longitudinal serum samples from patients with pneumonitis. Baseline: pre-I+N therapy (time point A), toxicity (time point C): at the time of pneumonitis. **(B)** NoirAE: Samples at 12 weeks of I+N therapy from patients with no irAEs (time point B). Pneumonitis: at the time of grade ≥2 pneumonitis (time point C). Same pneumonitis cases are represented in **(A, B)**, one patient with pneumonitis did not have baseline blood and, therefore, could not included in the longitudinal antibody titer figure in **(A)**.

Only the IgM antibody against ACHRG showed an increase from baseline to time of toxicity among pneumonitis cases (p=0.03, FDR= 0.48) and was also elevated during pneumonitis event as compared to 12-week “control” samples (p=0.00018, FDR= 0.012) (**Figure 3**).

### 3.4 Dermatitis

When samples obtained at the time of dermatitis were compared with 12-week samples from controls, IgM antibody against cytokeratin 19 antigen was higher in patients with dermatitis.

**Figure 4:**
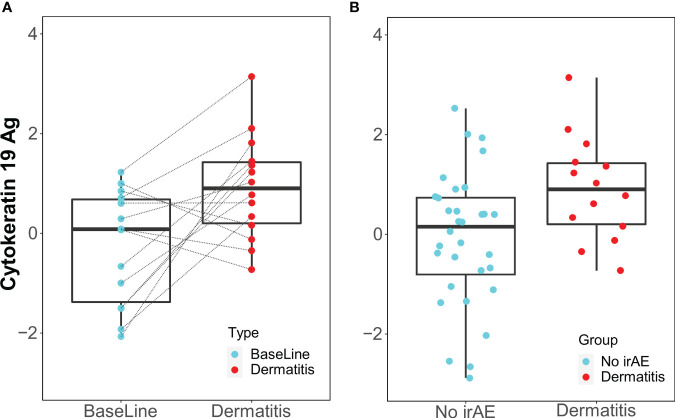
Box Plots of IgM antibody for Cytokeratin 19 Ag. **(A)** Longitudinal serum samples from patients with dermatitis. Baseline: pre-I+N therapy, toxicity: at the time of dermatitis. **(B)** NoirAE: Samples at 12 weeks of I+N therapy from patients with no irAEs. Dermatitis: at the time of grade ≥2 Dermatitis irAE.

Only the IgM antibody against cytokeratin 19 showed an increase from baseline to the time of toxicity among dermatitis cases (p=0.014, FDR=0.93) and was elevated during dermatitis events compared with the 12-week control samples (p=0.016, FDR=0.85) (**Figure 4**).

### 3.5 Diarrhea/colitis

None of the 120 auto-reactive antibodies in the IgG and IgM fractions were elevated at the time of toxicity compared to the 12-week control samples (**Figure 2B**).

### 3.6 Hepatitis

When samples obtained at the time of hepatitis were compared with 12-week samples from controls, IgG antibodies against thyroglobulin were higher at the time of toxicity compared to baseline samples among those who developed hepatitis. When samples at baseline were compared to samples at the time of toxicity IgG antibodies against TGF beta1, lactoferrin, and thyroglobulin were elevated compared to the 12-week control samples.

Only the IgG antibody against thyroglobulin was elevated during hepatitis events compared with the 12-week control samples (p=0.008, FDR=0.43) and showed an increase from baseline to the time of toxicity among hepatitis cases (p=0.036, FDR=0.84) (**Supplementary Figure 2**). IgM antibody against thyroglobulin did not show any statistically significant difference at the baseline among those who developed hepatitis, compared with baseline samples from patients who did not experience irAEs (p=0.16, FC (fold change): -1.5, FDR: 0.39); and at the time of hepatitis when compared with controls (p=0.354, FC: 1.4, FDR: 0.99).

IgG and IgM auto reactive antibodies for pneumonitis (**Supplementary Tables 4–6.1B**), dermatitis (**Supplementary Tables 6.2A, B**), diarrhea/colitis (**Supplementary Tables 6.3A, B**), hepatitis at baseline, at the time of toxicity (time of toxicity vs 12 week in normal controls and at the time of toxicity vs 12 week time point from patients with irAE) (pneumonitis: **Supplementary Table 7.1 A, B**; dermatitis **Supplementary Tables 7.2A, B**; diarrhea/colitis **Supplementary Tables 7.3A, B**, hepatitis **Supplementary Tables 7.4A, B**) with corresponding p values, fold change and false discovery rates and longitudinal changes for these toxicities (**Supplementary Tables 8.1A-8.8B**) provided in the supplement.

Subsequent sample collections following systemic steroid therapy were only available for four patients with dermatitis, where 3 out of 4 patients had a drop in Cytokeratin 19 auto reactive antibody titer with treatment (**Supplementary Figure 3**).

## 4 Discussion

In this study, we systematically evaluated the correlation between auto-reactive antibodies and irAEs in patients who had organ-specific toxicities. We observed that distinct auto-reactive antibodies were elevated at the time of toxicity in pneumonitis, dermatitis, and hepatitis.

Notably, IgM antibodies against ACHRG in pneumonitis, cytokeratin 19 during dermatitis, and IgG antibody against thyroglobulin during hepatitis were significantly elevated during toxicity events compared with 12-week control samples. These auto-reactive antibodies also showed an increase from baseline to the time of toxicity in patients with pneumonitis, dermatitis, and hepatitis respectively. Neither IgG nor IgM auto-reactive antibodies were elevated in diarrhea/colitis at the time of toxicity compared to the 12-week control samples.

Pneumonitis was associated with a unique auto-reactive antibody profile with elevations in multiple IgG and IgM auto-reactive antibodies, perhaps representing a marked increase in humoral autoimmunity. Some of these auto-reactive antibodies are particularly notable because they have been associated with lung injury outside of NSCLC. For example, antibodies that 1) have been implicated in lung allograft injury following a lung transplant (ACHRG); 2) are against soluble factors that play a role in lung inflammation (BAFF) (24, 25); 3) are against the cytoskeletal structure of the alveolar epithelium (cytokeratin 19); and 4) are elevated during tissue damage (NSE, SCCA, and Beta-glucuronidase) (26–31) have been noted to have higher titers in patients with pneumonitis than in patients without irAEs at the time of toxicity.

ACHRG, the cholinergic receptor nicotinic gamma subunit, plays a role in neuromuscular development (32). The antibodies targeting AChR (Acetylcholine receptors) are heterogenous in their reactivity with different subunits of the AChR. Previous studies reported that antibodies to adult or fetal Acetylcholine receptors (AChR) are negative in the healthy subjects but are sometimes positive in patients with myasthenia gravis (MG). One study reported among the 200 patients with MG, antibodies specific to ACHRG were detected in only 14 (7%) (33–35), indicating that the presence of these antibodies is not an incidental finding. Antibodies against ACHRG, have been previously reported (as AChR3) to be elevated in primary graft dysfunction (PGD) after lung transplant and was reported to be ≥ 2-fold higher in patients with PGD (36, 37). Nicotinic AChR is activated by nicotine and expressed in numerous non-neuronal cell types as well, including distinct populations of astrocytes, epithelial cells, adipocytes, lymphocytes, macrophages, keratinocytes, and stimulation of this receptor has well described anti-inflammatory effects (38–41). Other studies have associated multiple single-nucleotide nicotinic acetylcholine receptor polymorphisms with the risk of lung cancer and chronic obstructive pulmonary disease, highlighting their potential implications in respiratory diseases (42–44).

Cytokeratin 19 is a filament protein that is abundant in epithelial cells and has been overexpressed in non-small cell lung cancer, and its overexpression is correlated with a poor prognosis (45–47). In healthy individuals, while its level is low in circulation, it rises significantly in patients with epithelial cell-associated carcinomas (48). Similarly, antibodies against Cytokeratin 19 and its fragments can be observed in the majority of human serum samples (49–51). Its titers were reported to increase in epithelioid tumors such as lung cancer and patients with tissue injury such as alcoholic hepatitis and lung injury-related diseases such as pulmonary fibrosis and COPD compared to healthy controls (52–54).

Auto-reactive antibodies to thyroglobulin can be seen in healthy adults. In a population study of 17353 people, thyroglobulin antibody was detected in 11.5% of the study cohort (55). The positive antibodies were reported in up to 70-80% of patients with autoimmune thyroiditis, 30-40% with Graves disease, and 10-15% with non-thyroid autoimmune disease (56). Higher thyroglobulin antibody titers were reported in non-alcoholic liver disease and cirrhosis (57–59). In our study cohort, none of the patients who had elevated thyroglobulin IgG antibody levels during hepatitis had thyroid dysfunction at the time of the toxicity event or at the time of their subsequent follow-up.

Risk prediction and early diagnosis of irAEs are critical but also challenging for several reasons (60, 61). Many of the symptoms or irAEs can be difficult to distinguish from the disease process itself. For example, for immune-related pneumonitis, nonspecific clinical presentation with dyspnea, cough, and radiologic findings can mimic lung infection or lymphangitic spread, which may delay the diagnosis. Early detection and effective management of irAEs are critical to halt the progression to high-grade toxicities that are potentially life-threatening, but this generally entails constant clinical vigilance without effective diagnostic biomarkers. Auto-reactive antibody panels may be a practical adjunct tool for identifying at-risk patients, early diagnosis during treatment, and monitoring.

In our study, neither the IgG nor IgM auto-reactive antibodies analyzed in our panel were elevated in diarrhea/colitis; in fact, most of these auto-reactive antibodies had lower titers during toxicity events than the 12-week control samples. These findings highlight the potential risk of pooling multi-organ toxicities or analyzing overlapping toxicities when developing and validating novel biomarkers, since different aspects of the immune system may be driving the organ-specific toxicity. For example, during the course of colitis/diarrhea, the innate immune system may be playing a greater role in the development of organ-specific toxicity, similar to Crohn’s disease. But notably, pathology that underpins immune-related intestinal toxicities is driven by CD8 cytotoxic T lymphocytes, as has been shown in colonic biopsies (62–64). On the other hand, malabsorption and protein-losing enteropathy, severe anorexia, nausea, and vomiting may further exacerbate low antibody levels.

It is important to mention that a recent study suggested the role of baseline serum autoantibody signature in predicting toxicity in melanoma patients by using proteome microarray; this study focused on auto-reactive antibodies to the human proteome (65). In addition, previous work from our group using serological analysis of recombinant tumor cDNA expression libraries (SEREX) technology identified a number of autoimmune antibodies associated with pneumonitis and hypophysitis in patients with various types of cancer who received ICI therapy.

Our current study used a larger cohort of longitudinal samples, and a novel platform independently identified a larger number of auto-reactive antibodies that are associated with a wider spectrum of irAEs. Collectively, our data highlight the promising role of auto-reactive antibodies as predictive markers of immunotherapy-related toxicities.

This is a hypothesis-generating study with multiple strengths. In this study, samples were collected from a prospective clinical trial, all patients receiving same immunotherapy and toxicity attributions were made prospectively. Baseline samples were collected from immunotherapy-nave patients. Serial samples were available for comparative analysis and 12-week “control” samples were selected from patients who were observed for 6 subsequent months without the development of irAEs. Our auto-reactive antibody panel had a similar range of reportable results, reference intervals, reproducibility, and quality controls as other panels used in prior scientific studies (20, 66, 67). Finally, we used the false discovery rate to control the type I error rate associated with multiple comparisons and provided the FDR for each marker in the supplementary file.

There are gaps in knowledge on the pathogenesis of irAEs. Several mechanisms have been identified, including but not limited to cross-presentation of antigens, epitope spreading, autoantibody production, inflammatory monocyte activation, complement-mediated inflammation, inflammatory cytokines, host-specific factors including microbiome and genetics, and the type of ICI immunotherapy administered (68–70) (Ibis, Aliazis), and this study is only focusing in humoral immune systemic and autoantibody production. Therefore, it does not provide a comprehensive assessment of the immune system. This study has some additional limitations. First, it had a relatively limited sample size and lacked an independent validation cohort. Second, we could not confirm the cutoffs that would indicate a clinically significant elevation in auto-reactive antibody titers. Third, our auto-reactive antibody panel may not have included all relevant targets. For example, anti-CD74 antibodies were associated with pneumonitis in our previous study, but this was not present in our panel (8). Finally, we cannot determine whether auto-reactive antibodies are the result of tissue damage or responsible for tissue damage, i.e. whether they have a causal role in the development of irAEs. Further studies are needed to investigate the mechanism whereby auto-reactive antibodies result in irAEs.

In summary, we report that several auto-reactive antibodies are elevated at baseline and during toxicities, such as pneumonitis, dermatitis, and hepatitis. In particular, the IgM fraction of auto-reactive antibodies against ACHRG in pneumonitis, cytokeratin 19 in dermatitis, and IgG antibody against thyroglobulin during hepatitis were elevated during the time of toxicity compared with 12-week control samples and showed an increase from baseline to the time of toxicity among pneumonitis, dermatitis, and hepatitis cases, respectively, and should be investigated further. Future studies are warranted to validate these findings and explore the mechanistic relationships of these antibodies and their potential roles in the toxicity and early recognition of irAEs.

## Data availability statement

The original contributions presented in the study are included in the article/**Supplementary Material**, further inquiries can be directed to the corresponding author/s.

## Ethics statement

The studies involving humans were approved by University of Texas MD Anderson Cancer Center Institutional Review Board. The studies were conducted in accordance with the local legislation and institutional requirements. The participants provided their written informed consent to participate in this study.

## Author contributions

MA: Conceptualization, Data curation, Formal analysis, Funding acquisition, Investigation, Methodology, Project administration, Supervision, Writing – original draft. QL: Data curation, Formal analysis, Methodology, Resources, Writing – review & editing. QW: Data curation, Formal analysis, Writing – review & editing. NV: Investigation, Writing – review & editing. AS: Writing – review & editing. JG: Writing – review & editing. CZ: Data curation, Formal analysis, Writing – review & editing. HT: Investigation, Resources, Writing – review & editing. SG: Investigation, Writing – review & editing. MA: Investigation, Writing – review & editing. SS: Writing – review & editing. JW: Data curation, Writing – review & editing. LB: Investigation, Writing – review & editing. NA: Writing – review & editing. MF: Writing – review & editing. YW: Writing – review & editing. JL: Writing – review & editing. JZ: Investigation, Methodology, Writing – review & editing. JH: Conceptualization, Funding acquisition, Investigation, Methodology, Resources, Supervision, Writing – review & editing.
