# Supplementary material for: Distinct patterns of auto-reactive antibodies associated with organ-specific immune-related adverse events
Source: Front Immunol. 2023 Dec 12;14:1322818. doi: 10.3389/fimmu.2023.1322818 (PMC10751952; doi:10.3389/fimmu.2023.1322818)
Supplement: Supplementary file 1 [file DataSheet_1.pdf]

SUPPLEMENTARY APPENDIX:

Distinct patters of Auto-Reactive Antibodies associated with organ specific Immune-Related Adverse Events

Contents

Supplementary methods: ... 4

1) STUDY SYNOPSIS: ... 4

Supplemental figure 1: Study schema ... 4

2) AUTOANTIGENS INCLUDED IN MICROARRAY: ... 9

Supplementary Table 1: List of Auto reactive antibodies ... 9

3) BATCH CORRECTION FORMULA: ... 12

Supplemental table 2 A and B ... 12

4) CLINICAL CHARACTERISTICS: ... 13

Supplementary Table 3. Clinicopathologic features of patients with/out irAE ... 13

5) IG G AND M AUTOREACTIVE ANTIBODIES IN PATIENTS WITH PNEUMONITIS ... 15

Supplementary Table 4. IgG autoreactive antibodies in patients with pneumonitis at the time of toxicity ... 15

Supplementary table 5. List of IgM Auto reactive antibodies elevated at baseline and at the time of pneumonitis event ... 15

6) FALSE DISCOVERY AND P VALUES FOR AUTO REACTIVE ANTIBODIES AT BASELINE ... 17

Supplementary tables 6.1.A. and 6.1.B. Supplementary tables for PNEUMONITIS, baseline auto reactive antibody panel comparison for pneumonitis vs. non-irAE group, Ig G and Ig M**:** ... 17

Supplementary table 6. 1.A) Ig G ... 17

Supplementary table 6.1.B) IgM ... 17

Supplementary tables 6.2.A. and 6.2.B. Supplementary tables for DERMATITIS, baseline auto reactive antibody panel comparison for dermatitis vs. non-irAE group, Ig G and Ig M: ... 19

Supplementary table 6.2.A. IgG ... 19

Supplementary table 6.2.B. IgM ... 19

Supplementary tables 6.3.A. and 6.3.B. DIARRHEA/COLITIS, baseline auto reactive antibody panel comparison for diarrhea/colitis vs. non-irAE group, Ig G and Ig M: ... 20

Supplementary tables 6.3.A. Ig G ... 20

Supplementary tables 6.3.B. IgM ... 20

Supplementary tables 6.4.A. and 6.4.B. HEPATITIS, baseline auto reactive antibody panel comparison for hepatitis vs. non-irAE group, Ig G and Ig M: ... 20

Supplementary table 6.4.A. Ig G ... 20

Supplementary table 6.4.B. Ig M 20
7) FALSE DISCOVERY AND P VALUES FOR AUTO REACTIVE ANTIBODIES AT TOXICITY 21
Supplementary tables 7.1.A. and 7.1.B. Antibody panel comparison for pneumonitis, at the time of toxicity event vs. 12-week control blood from non-irAE group........ 21
Supplementary table 7.1.A. IgG 21
Supplementary table 7.1.B. Ig M 22
Supplementary tables 7.2.A. and 7.2.B. Antibody panel comparison for dermatitis, at the time of toxicity event vs. 12 week control blood from non-irAE group 24
Supplementary table 7.2.A. Ig G 24
Supplementary table 7.2.B. IgM 24
Supplementary tables 7.3.A. and 7.3.B. Antibody panel comparison for diarrhea/colitis, at the time of toxicity event vs. 12-week control blood from non-irAE group........ 24
Supplementary table 7.3.A. 24
Supplementary table 7.3.B........ 24
Supplementary tables 7.4.A. and 7.4.B. Antibody panel comparison for hepatitis, at the time of toxicity event vs. 12-week control blood from non-irAE group........ 24
Supplementary table 7.4.A. IgG 24
Supplementary table 7.4.B. IgM 24
8) LONGITUDINAL CHANGES FOR AUTO REACTIVE ANTIBODIES IN TOXICITY GROUP WITH FALSE DISCOVERY AND P VALUES 25
Supplementary tables 8.1.A. and 8.1.B. Antibody panel comparison at baseline and at the time of toxicity for pneumonitis........ 25
Supplementary table 8.1.A. IgG 25
Supplementary table 8.1.B. IgM 25
Supplementary tables 8.2.A. and 8.2.B. Antibody panel comparison at baseline and at the time of toxicity for dermatitis 25
Supplementary table 8.2.A. IgG 25
Supplementary table 8.2.B. IgM 26
Supplementary tables 8.3.A. and 8.3.B. Antibody panel comparison at baseline and at the time of toxicity for diarrhea/colitis 26
Supplementary table 8.3.A. IgG 26
Supplementary table 8.3.B. Ig M 26
Supplementary tables 8.4.A. and 8.4.B. Antibody panel comparison at baseline and at the time of toxicity for hepatitis........ 26
Supplementary table 8.4.A. Ig G 26

Supplementary table 8.4.B. Ig M 26
Supplementary tables 8.5.A. and 8.5.B. Antibody panel comparison at baseline and at the end of induction for pneumonitis 27
Supplementary table 8.5.A IgG 27
Supplementary table 8.5.B. IgM 27
Supplementary tables 8.6.A. and 8.6.B. Antibody panel comparison at baseline and at the end of induction for dermatitis 27
Supplementary table 8.6.A IgG 27
Supplementary table 8.6.B. IgM 27
Supplementary tables 8.7.A. and 8.7.B. Antibody panel comparison at baseline and at the end of induction for diarrhea/colitis 28
Supplementary table 8.7.A IgG 28
Supplementary table 8.7.B. IgM 28
Supplementary tables 8.8.A. and 8.8.B. Antibody panel comparison at baseline and at the end of induction for hepatitis 28
Supplementary table 8.8.A IgG 28
Supplementary table 8.8.B. IgM 28
9) LONGITUDINAL CHANGES FOR AUTO REACTIVE ANTIBODIES IN NO TOXICITY GROUP WITH FALSE DISCOVERY AND P VALUES 29
Supplementary tables 9.A. and 9.B. Antibody panel comparison at baseline and in 12 weeks in no irAE group 29
Supplementary table 9.1.A. Ig G 29
Supplementary table 9.1.B. Ig M 30
10) IgG ANTIBODY FOR THYROGLOBULIN IN HEPATITIS 32
Supplemental Figure 2. Box plots for IgG antibody for thyroglobulin 32
11) AUTO REACTIVE ANTIBODY TITERS FOLLOWING STEROID USE: 33
Supplemental figure 3. Longitudinal Cytokeratin 19 IgM titers in patients with dermatitis 33
REFERENCES 33

Supplementary methods:

1) STUDY SYNOPSIS:

NCT 03391869 study synopsis.pdf

Major Inclusion Criteria: 1) Histologically confirmed NSCLC, 2) AJCC 8th edition stage IV disease, 3) EGFR wild type and no ALK fusion, 4) candidate for immunotherapy, and no prior history of this treatment; note that one prior course of chemotherapy is allowed, 5) candidate for radiation therapy to at least one site of disease.

Study Schema: Patients who do not progress after 12 weeks of treatment with ipilimumab/nivolumab will be randomized in a 1:1 fashion to: a) continuation of ipilimumab/nivolumab until progression or up to 2 years, vs. b) LCT, defined as surgery or radiation, with radiation to at least one site of disease plus ipilimumab/nivolumab until progression or up to 2 years. Surgery on the primary site will be strongly encouraged if feasible. For patients with oligometastatic disease, LCT will encompass all sites of disease. For patients with >3 lesions (polymetastatic disease), LCT will be performed on as many sites as the treating physician deems to be feasible and with acceptable tolerance (supplemental figure 1).

Supplemental figure 1: Study schema

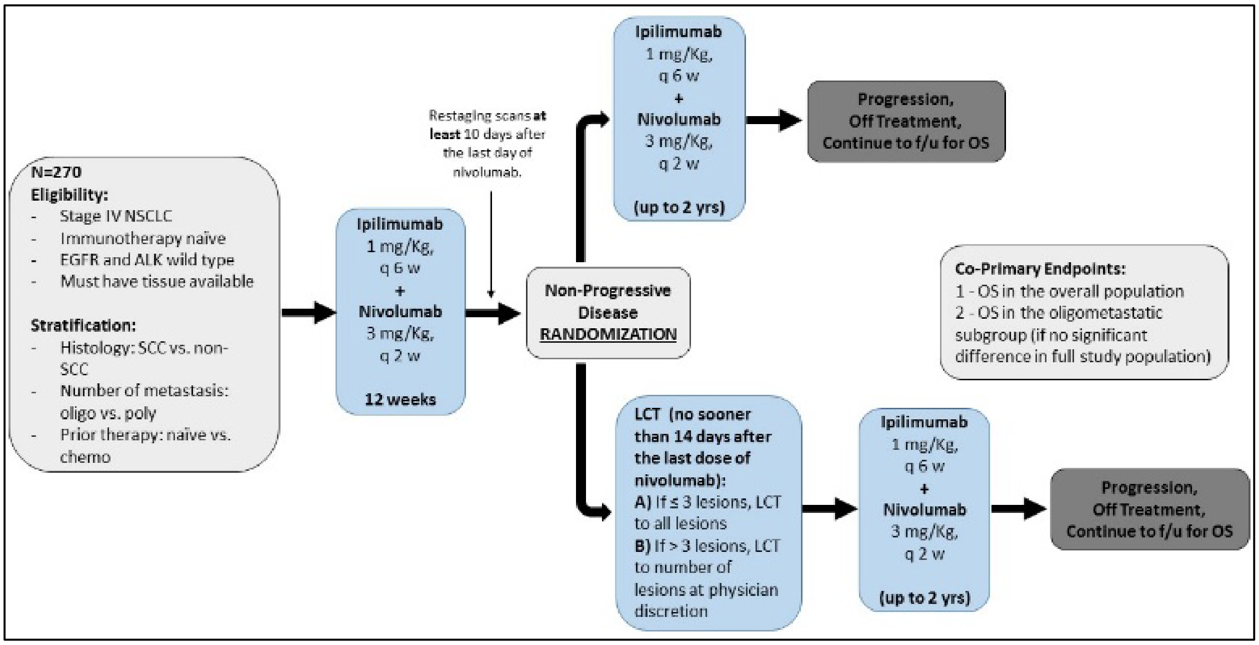

Statistical Considerations: Based on prior data from the Checkmate 017 and 057 (2, 3), as well as the PFS time for patients on first line systemic therapy, we estimated the OS time in the standard (ipilimumab/nivolumab) cohort to be 14.0 months. Based on data from our recently published

oligometastatic study, the OS time in this population was estimated to be 16.0 months. Sample size and effect size estimates are as follows:

A) OS assessment for the entire cohort (combined oligometastasis and polymetastasis).

• We plan to test the null hypothesis of no difference OS between patients randomized to LCT+ ipilimumab/nivolumab versus ipilimumab/nivolumab alone.

• Ho: $\rho = 1.0$ versus Ha: $\rho < 0.68$; where ρ represents the hazard ratio (HR).

• A HR = 0.68, corresponding to a median OS benefit from 14 months to 20.6 months.

• A sample size of 216 randomized patients (108 patients per study arm) will yield approximately 80% power to detect a HR = 0.68 using a 1-sided log-rank test with a significance level of 0.05. This aspect of study requires a maximum of 178 events.

• The sample size is inflated (to account for 40% nonrandomization rate) to 360 patients due to potential dropouts from progression or toxicity during front-line immunotherapy (Or similarly, if approximately 40% of patients are expected to have a progression or toxicity during front-line immunotherapy; then 216/0.60 = 360 patients are required).

B) OS assessment for the oligometastatic cohort (to be done as primary endpoint if: a) 1A the null hypothesis (described above) is not rejected, or b) 1B as part of the futility analysis if stopping criterion for futility is met).

• We plan on randomizing a minimum of 74 patients with oligometastatic disease regardless of the outcome of the futility analysis, as noted above. Accruing a larger proportion of patients with oligometastatic disease during the study period may not be feasible given the number of patients that are seen at MD Anderson on a yearly basis. However, completing the enrollment will ensure that a sufficient number of patients are accrued to make the desired statistical comparisons between groups. A sample size of 74 patients with oligometastatic disease will provide 37 patients randomized to each study arm: LCT+ ipilimumab/nivolumab versus ipilimumab/nivolumab alone.

• A sample size of 74 patients yield 80% power with a 1-sided significance level of 0.05 to detect a HR = 0.51 using a 1-sided log-rank test with a significance level of 0.05.

• A HR = 0.51, corresponding to a median OS benefit from 16 months to 31.4 months and will require a maximum of 55 events.

Accrual Goal and Rate for the Study: The total patients to be accrued is 360 to ensure at least 216 patients are randomized (of which a minimum of 74 patients with oligometastatic disease will be acquired).

<u>Inclusion Criteria</u>

1) Age > 18

2) Histologically or cytologically confirmed non-small cell lung cancer. If a diagnostic biopsy is available, a pre-treatment biopsy is not required. Patients with a suspected lung cancer may be consented, but pathology must be confirmed prior to initiating treatment on study. Neuroendocrine carcinomas (e.g. SCLC, carcinoid tumors) are not eligible. Carcinomas with neuroendocrine differentiation are eligible.

3) Stage IV (according to AJCC 8th edition) measurable disease per RCESIST 1.1.

4) Signed and dated written informed consent prior to admission to the study in accordance with ICH-GCP guidelines and to the local legislation.

5) For lung adenocarcinoma patients, patients must not harbor any EGFR sensitizing mutations or ALK fusion where there are standard of care therapy options available. For patients with histologies other than adenocarcinoma, EGFR and ALK status is not required. Adenocarcinoma patients may be consented prior to the EGFR and ALK status being known, but EGFR and ALK status must be determined prior to initiating therapy. EGFR and ALK status may be determined using either tumor- or plasma-based, CLIA-certified assays. For patients with NSCLC, not otherwise specified (NOS), EGFR/ALK testing is not required, as the frequency of alterations is exceedingly rare in this histology. Also, note that patients with ROS1 or RET alterations can be enrolled, as Tyrosine inhibitor such crizotinib aren’t established as first line therapy for patients with these alterations.

6) One prior line of chemotherapy and/or targeted agents for metastatic disease are permitted. This chemotherapy can include maintenance therapy, as long as it was given in the front line setting. In addition, prior antiangiogenic therapy (e.g. bevacizumab) is permitted if used as frontline treatment.

7) Patients must have organ and marrow function as defined below:

➢ Performance Status of 0 or 1 if using ECOG/Zubrod.

➢ Screening laboratory values must meet the following criteria and should be obtained within 14 days prior to treatment initiation

➢WBC≥ 2000/µL

➢Neutrophils≥ 1500/µL

➢ Platelets≥ 100 x10^3/µL

➢ Hemoglobin > 9.0 g/dL

➢ Serum creatinine ≤ 1.5 x ULN or creatinine clearance (CrCl) ≥ 50 mL (if using the Cockcroft-Gault formula below):

➢ Female CrCl = (140 - age in years) x weight in kg x 0.85 72 x serum creatinine in mg/dL

➢ Male CrCl = (140 - age in years) x weight in kg x 1.00 72 x serum creatinine in mg/dL

➢ AST/ALT ≤ 3 x ULN

➢ Total Bilirubin ≤ 1.5 x ULN (except subjects with Gilbert Syndrome, who can have total bilirubin < 3.0 mg/dL)

8) Women of childbearing potential (WOCBP) must use appropriate method(s) of contraception. Appropriate methods of contraception are as follows. Women will be instructed to adhere to contraception for a period of 26 weeks after the last dose of investigational product. Men receiving

nivolumab and who are sexually active with WOCBP will be instructed to adhere to contraception for a period of 35 weeks after the last week of nivo/ipi.

Note: WOCBP is defined as any female who has experienced menarche and who has not yet undergone surgical sterilization (hysterectomy or bilateral oophorectomy) or who is not postmenopausal. Menopause is defined clinically as 12 months of amenorrhea in a woman over 45 in the absence of other biological or physiological causes. In addition, women under the age of 55 must have a documented negative serum or urine test.

9) Women of childbearing potential must have a negative serum or urine pregnancy test within 48 hours prior to the start of nivolumab.

10) Men who are sexually active with WOCBP must use any contraceptive method with a failure rate of less than 1% per year. Men receiving nivolumab and who are sexually

active with WOCBP will be instructed to adhere to contraception for a period of 35 weeks after the last dose of investigational product Women who are not of childbearing potential (ie, who are postmenopausal or surgically sterile as well as azoospermic men do not require contraception.

11) Subjects with brain metastases are eligible if metastases are adequately treated and subjects are neurologically stable (except for residual signs or symptoms related to the CNS treatment) for at least 2 weeks prior to the first dose of nivolumab. In addition, subjects must be either off corticosteroids, or on a stable or decreasing dose of ≤ 10 mg daily prednisone (or equivalent) for at least 2 weeks prior to the first dose of nivolumab. Patients with asymptomatic, small (e.g. ≤1 cm) brain metastases are eligible provided that the patient is off corticosteroids, or on a stable or decreasing dose of ≤ 10 mg daily prednisone (or equivalent) for at least 2 weeks prior to the first dose of nivolumab.

12) Subjects may receive radiotherapy for symptomatic metastases prior to enrollment provided that there is at least one other non-irradiated lesion amenable to LCT at the time of enrollment. When feasible, stereotactic body radiation therapy (SBRT) or other

hypofractionated techniques are strongly encouraged.

Exclusion Criteria

1) Systemic immunotherapy for metastatic NSCLC. Immunotherapy agents include, but are not limited to, agents targeting the PD1/PD-L1 axis (e.g. nivolumab, pembrolizumab, atezolizumab, durvalumab) or CTLA-4 (ipilimumab, tremelimumab) pathways.

2) Any approved anti-cancer therapy, including chemotherapy, or hormonal therapy within 3 weeks prior to the initiation of study treatment; the following exceptions are allowed:

o Hormone-replacement therapy or oral contraceptives

3) Women must not be breastfeeding.

4) Patients excluded with prior treatment of pneumonitis requiring corticosteroids within 60 days prior to the first dose of nivolumab.

5) Unwillingness or inability to follow the procedures required in the protocol.

6) Any serious or uncontrolled medical disorder that, in the opinion of the investigator, may increase the risk associated with study participation or study drug administration, impair the ability of the subject to receive protocol therapy, or interfere with the interpretation of study results.

7) Prior malignancy active within the previous 2 years. Patients with locally curable cancers that have been apparently cured, such as basal or squamous cell skin cancer, superficial bladder cancer, or carcinoma in situ of the prostate, cervix, or breast with local control measures (surgery, radiation) are eligible.

8) Patients should be excluded if they have an active, known or suspected autoimmune disease. Subjects are permitted to enroll if they have vitiligo, type I diabetes mellitus, residual hypothyroidism due to autoimmune condition only requiring hormone replacement, psoriasis not requiring systemic treatment, or conditions not expected to recur in the absence of an external trigger.

9) Patients should be excluded if they have a condition requiring systemic treatment with either corticosteroids (> 10 mg daily prednisone equivalents) or other immunosuppressive medications within 14 days of study drug administration (i.e. disease-modifying antirheumatic drugs). Inhaled or topical steroids and adrenal replacement doses > 10 mg daily prednisone equivalents are permitted in the absence of active autoimmune disease.

Note that subjects are permitted to use topical, ocular, intra-articular, intranasal, and inhalational corticosteroids (with minimal systemic absorption). Physiologic replacement doses of systemic corticosteroids are permitted, even if >10 mg/day prednisone equivalents. A brief course of corticosteroids for prophylaxis (e.g. contrast dye allergy) or for treatment of non-autoimmune conditions (e.g. delayed- type hypersensitivity reaction caused by contact allergen) is permitted.

10) As there is potential for hepatic toxicity with nivolumab or nivolumab/ipilimumab

combinations, drugs with a predisposition to hepatoxicity should be used with caution.

11) Patients should be excluded if they are known to be positive for hepatitis B virus surface antigen (HBV sAg) or hepatitis C virus ribonucleic acid (HCV antibody) indicating acute or chronic infection.

12) Patients should be excluded if they have known history of testing positive for human immunodeficiency virus (HIV) or known acquired immunodeficiency syndrome (AIDS).

13) History of allergy to study drug components.

14) History of severe hypersensitivity reaction to any monoclonal antibody.

15) Prisoners or subjects who are involuntarily incarcerated.

16) Subjects who are compulsorily detained for treatment of either a psychiatric or physical (infection disease) illness.

17) Psychological, familial, sociological or geographical factors potentially hampering compliance with the study protocol and follow-up schedule.

18) Any condition that, in the opinion of the investigator, would interfere with the study treatment or interpretation of the study results.

2) AUTOANTIGENS INCLUDED IN MICROARRAY:

All of the list of auto reactive antibodies, their description and vendor information is available in Supplemental table 2. Further information is also available at genomics and microarray core website https://genomics-microarray.swmed.edu and list of publications at https://genomics-microarray.swmed.edu/staff/publications/

Supplementary Table 1: List of Auto reactive antibodies

Auto antibody	Auto Antibody decription	*Vendor*
AChR3	Acetylcholine receptor subunit gamma Recombinant Protein	MyBiosource.com
AFP	alpha-fetoprotein	Bio-Rad
ALDOC	Aldolase C	Genway
Aldolase (muscle)	Aldolase (muscle)	Sigma-Aldrich
α-2-macroglobulin-like 1	Alpha-2-macroglobulin-like 1	R&D Systems
Alpha-fodrin	Alpha-fodrin	cloud-clone corp.
AGTR1	Angiotensin II Receptor Type 1	MyBiosource.com
ANXA1	Annexin A1	Novoprotein
Apolipoprotein B/E	Apolipoprotein B and E	Sigma
Azurocidin	Azurocidin	Meridian Life Science, Inc.
BAFF	B-cell activating factor	Biolegend
Beta-glucuronidase	beta glucuronidase	Sigma
BRCA1 and BRCA2	Breast Cancer gene 1 and 2	abnova
CA125	Cancer Antigen 125	Fitzgerald
CA15-3	Cancer Antigen 15-3	LEE
CA19-9	Cancer Antigen 19-9	Meridian Life Science, Inc.
CA242	Cancer Antigen 242	Creative Biomart
CA27-29	Cancer Antigen 27-29	Fitzgerald
CA50	Cancer Antigen 50	LEE
CA72-4	Cancer Antigen 72-4	Fitzgerald
Calmodulin (brain)	Calmodulin (brain)	Calbiochem
Calreticulin(rabit calreticulin)	Calreticulin(rabit calreticulin)	Sigma Aldrich
Carbonic Anhydrase 6 (CA6)	Carbonic Anhydrase 6 (CA6)	R & D systems
Catalase	Catalase	Sigma
Cathepsin G	Cathepsin G	Enzyme Research Laboratories
CCL11	C-C motif chemokine 11	BioLegend
CCL2	C-C motif chemokine 2	BioLegend
CCL3	C-C motif chemokine 3	BioLegend

CCL5	C-C motif chemokine 5	BioLegend
CDK2	Cycline Dependent kinase 2	Life Technology
CEA	Carcinoembryonic antigen	Fitzgerald
Cholinergic Receptor Muscarinic 3 (CHRM3)	cholinergic receptor muscarinic 3	Abnova
CMV (G and M)	Cytomegalovirus Ig G and Ig M	Meridian Life Science, Inc.
CMV EXT (EXT-2??)	Cytomegalovirus	Meridian Life Science, Inc.
CMV GRADE III ANTIGEN	Cytomegalovirus Grade III Antigen	Meridian Life Science, Inc.
C-MYC	cellular-myc	Abcam
CTLA4	cytotoxic T-lymphocyte–associated antigen 4	Biolegend
CXCL10	C-X-C motif chemokine ligand 10	Biolegend
Cytokeratin 19 Ag	Cytokeratin 19 Ag	Biorad
DDX53	DEAD-Box Helicase 53	Abnova
dsDNA	Double stranded DNA	DIARECT
EBV EBNA1	EBV/EBNA1	Syd Labs
EGF Receptor	Epidermal growth factor receptor	Prospec-Tany-Technogene
Elastase	Elastase	Promega
Endothelial Cell Extract	Endothelial Cell Extract	Sciencellonline.com
Elastase	Elastase	Enzo life science
ErbB2	Erb-B2 Receptor Tyrosine Kinase 2	Biovision
ErbB3 and ErbB4	Erb-B2 Receptor Tyrosine Kinase 3 and 4	Biorbyt
ERP29	Endoplasmic Reticulum Protein 29	LSBio
Ferritin	Ferritin	Cell Sciences
Flagellin CBir1	Anti-flagellin	Biorbyt
FUCA1	Alpha-L-Fucosidase 1	OriGene Technologies
Galactocerebroside	Galactocerebroside	MyBiosource.com
GBM	glomerular basement membrane	DIARECT
GBU4-5 (TDRD-12)	Tudor domain-containing 12	Raybiotech
genomic DNA	genomic DNA	Roche
Glutamate decarboxylase-65/GAD2	Glutamic acid decarboxylase	antibodies-online
Hepatitis A antigen	Hepatitis A antigen	Meridian Life Science, Inc.
HPV E6 (16)	Human papillomavirus E6	Protein X Lab
HPV E7 (16+18)	Human papillomavirus E7	Protein X Lab
HSV-1 and HSV-2 antigen	Herpes simplex virus 1 and 2	Meridian Life Science, Inc.
HuD/ELAVL4	ELAV-like protein 4	Clonegene LLC
IFN α and β	Interferon alpha and beta	BioLegend
IFN-ɣ	Interferon gamma	BioLegend
IL-1 alpha and beta	Interleukin 1 alpha and beta	BioLegend
IL-12 (p70)	Interleukin-12	BioLegend
IL-17A	Interleukin-17A	Biolegend
IL-2	Interleukin-2	Biolegend

IL-6	Interleukin-6	Biolegend
IL-8	Interleukin-8	Biolegend
Lactoferrin	Lactoferrin	AbD Serotec
LC1	Liver cytosol type 1	DIARECT AG
LAMP-2	Lysosomal Associated Protein 2	DIARECT AG
MAG	Myelin-associated glycoprotein	LifeTech
MAGEA3	Melanoma-associated antigen A 3	Genway
MAGEA4	Melanoma-associated antigen A 4	Novus
MBP	Myelin basic protein (MBP)	EMD Millipore
MMP-2	Metalloproteinase-2	BioLegend
Muscarinic 3/CHRM3	Cholinergic Receptor Muscarinic 3	Abnova
NSE	Neuron-specific enolase	LEE
Nucleosome	Nucleosome	Arotec
NY-ESO-1	New York Esophageal Squamous Cell Carcinoma-1	Themo (Pierce)
OmpC	Outer membrane protein C	MyBiosource.com
Ox40L	Ox40L	BioLegend
P53	Tumor protein 53	Enzo life science
PSP	Parotid Secretory Protein	R&D Systems
PD-1	Programmed cell death protein 1	Abcam
PD-L1	Programmed cell death protein-ligand 1	Biorbyt
Peptidyl Arginine Deiminases 1-4	Peptidyl Arginine Deiminases 1-4	SignalChem
Phosphatidyl-l-serine	Phosphatidyl-l-serine	Sigma-Aldrich
PKM2	Pyruvate kinase M 1/2	Creative Biomart
PSA	Prostate Specific Antigen	Fitzgerald
PSMA	Prostate Specific Membrane Antigen	Fitzgerald
Prostatic Acid Phosphatase	Prostatic Acid Phosphatase	abcam
rhHSPG2	Recombinant Heparan Sulfate Proteoglycan 2	Abcam
Ribo Phospha Protein P0 P1 P2	Ribo Phospha Protein P0, P1 and P2	DIARECT
Ro-SSA (52 + 60)	Anti–Sjögren's-syndrome-related antigen A	DIARECT
Rotavirus SA-11	Simian rotavirus	Meridian Life Science, Inc.
RSV antigen	Respiratory syncytial virus antigen	Meridian Life Science, Inc
RSVP antigen	Rubella spike viral protein antigen	Meridian Life Science, Inc
Rubella virus grade III and IV antigen	Rubella virus grade III and IV antigen	Meridian Life Science, Inc
Rubeola antigen	Rubeola antigen	Meridian Life Science, Inc
Saccharomyces Cerevisiae	Saccharomyces Cerevisiae	R & D systems
SCCA	Squamous cell carcinoma antigen	Creative Biomart
Sm/RNP	Sm/RNP	DIARECT
SOX2	SRY-box 2	Primorigen
Sphingomyelin	Sphingomyelin	Sigma-Aldrich
Tetanus toxoid	Tetanus toxoid	Sigma-Aldrich

TGF-β1	Transforming growth factor- beta 1	Biolegend
TPO	Thrombopoietin TPO	DIARECT AG
Thyroglobulin	Thyroglobulin	DIARECT AG
TTG	Tissue Transglutaminase	DIARECT AG
TNF-α and β	Tumor necrosis factor-alpha and beta	Biolegend
Toxoplasma	Toxoplasma	Meridian Life Science
Troponin I	Troponin I	Meridian Life Science
Troponin I-T-C ternary complex mixture	Troponin I-T-C ternary complex mixture	Meridian Life Science, Inc
TWEAK (CD255)	TNF-like weak inducer of apoptosis	Biolegend
uPA	u-Plasminogen Activator	Abcam
VEGF-165	Vascular endothelial growth factor 165 protein	BioLegend
VZV antigen	Varicella-zoster virus	Meridian Life Science, Inc.

Autoantibody controls
Human IgG control
Human IgM control
anti-human IgG control
anti-human IgM control

3) BATCH CORRECTION FORMULA:

After the log2 transformation, the mean of the feature j across all baseline samples within batch k were calculated as shown in table 1 A, where n_k is the number of baseline/control samples in batch k. Then, the batch corrected value x^*_{ijk} for all samples was adjusted by where N_k is the number of total samples in batch k. Modified from the mean centering batch correction method[1], shown in table 2 B.

Supplemental table 2 A and B

Table 2 A	$\bar{x}_{jk} = \frac{1}{n_k}\sum_{c=1}^{n_k} x_{cjk},$

Table 2 B	$x^*_{ijk} = x_{ijk} - \bar{x}_{jk}, i = 1, 2, \ldots, N_k,$

4) CLINICAL CHARACTERISTICS:

Supplementary Table 3. Clinicopathologic features of patients with/out irAE

Age	Sex	Smoking hx	Prior chemo (Y: yes/ N: no)	Histopath	irAE 1	Grade	Time (days) from first dose of I+N to irAE1	If any other irAE	irAE 2	irAE 2 grade	timing of irAE (overlap/seperate)	time from first dose of I+N to irAE 2
70	M	Former	N	Adeno	diarrhea/colitis	3	267					
53	F	Former	Y	Large Cell	diarrhea/colitis	3	422					
60	M	Former	Y	Adeno	pneumonitis	2						
63	F	Former	N	SCC	No tox control							
85	M	Former	N	SCC	diarrhea/colitis	2	21					
47	M	Never	N	SCC	No tox control							
72	M	Former	N	Adeno	diarrhea/colitis	2	22	yes	nephritis	2	separate	91
64	M	Former	N	Adeno	dermatitis	2	84	yes	Arthritis/Arthralgia	2	separate	172
59	M	Never	N	NOS	No tox control							
71	M	Former	N	Adeno	pneumonitis	2	126	yes	Artritis/Arthralgia	2	separate	215
78	F	Former	N	SCC	pneumonitis	3	381					
70	M	Former	Y	Adeno	No tox control							
66	M	Current	N	SCC	dermatitis	2	91					
45	M	Never	N	Adeno	No tox control							
64	M	Former	Y	Adeno	No tox control							
54	F	Never	Y	Adeno	hepatitis	2	118					
54	F	Never	Y	Adeno	No tox control							
76	M	Former	N	Adeno	No tox control							
62	F	Former	Y	Adeno	dermatitis	2						
67	M	Former	N	SCC	No tox control							
69	F	Former	N	Adeno	diarrhea/colitis	3	28					
57	F	Former	N	Adeno	No tox control							
82	M	Former	N	SCC	No tox control							
53	M	Former	N	Adeno	diarrhea/colitis	3	75					
73	M	Former	N	Adeno	No tox control							
83	M	Former	N	SCC	hepatitis	2	161					
68	F	Former	Y	SCC	diarrhea/colitis	3	24	yes	Arthritis/Arthralgia	2	separate	182
71	F	Former	N	SCC	diarrhea/colitis	2						
74	M	Former	Y	Adeno	pneumonitis	2						
58	F	Former	N	Adeno	pneumonitis	2						
70	F	Current	N	SCC	diarrhea/colitis	2	92					
80	M	Former	N	Adeno	pneumonitis	3						
72	F	Former	N	Adeno	diarrhea/colitis	3	70	yes	Arthritis/Arthralgia	3	separate	203

54	F	Current	Y	Adeno	diarrhea/colitis	2	21	yes	adrenal insufficiency	3	separate	73
56	M	Current	N	SCC	pneumonitis	2	457					
62	M	Current	Y	SCC	No tox control							
85	M	Former	N	SCC	dermatitis	2	405					
69	F	Former	N	NOS	dermatitis	2	28					
59	M	Former	N	Adeno	No tox control							
63	F	Former	N	Adeno	pneumonitis	2	196					
59	F	Never	N	Adeno	pneumonitis	3	66					
75	M	Former	N	Adeno	dermatitis	2	82					
70	F	Former	N	Adeno	dermatitis	2	147					
65	M	Former	N	Adeno	diarrhea/colitis	2	77	yes	hepatitis	3	separate	122
84	M	Never	N	Adeno	No tox control							
61	F	Current	N	SCC	dermatitis	2	131					
64	F	Current	N	SCC	dermatitis	2	28	yes	diarrhea/colitis	2	separate	65
68	F	Former	Y	Adeno	hepatitis	2						
59	F	Former	Y	Adeno	diarrhea/colitis	3	77					
79	F	Never	N	NOS	dermatitis	2	89					
59	F	Former	N	Adeno	hepatitis	3	56					
77	F	Former	N	Adeno	dermatitis	2	90					
66	M	Never	N	Adeno	No tox control							
47	F	Current	N	Adeno	No tox control							
75	F	Former	N	NOS	pneumonitis	2	84					
72	F	Former	N	Adeno	hepatitis	3	49					
83	F	Former	N	Adeno	diarrhea/colitis	2	63					
49	F	Never	N	Adeno	dermatitis	2	70					

Clinical characteristics of the patients, details of the irAE event summarized in supplemental table 3.

5) IG G AND M AUTOREACTIVE ANTIBODIES IN PATIENTS WITH PNEUMONITIS

Supplementary Table 4. IgG autoreactive antibodies in patients with pneumonitis at the time of toxicity

Elevated IgG auto reactive antibodies at baseline	Elevated IgG auto reactive antibodies both at baseline and pneumonitis	Elevated IgG auto reactive antibodies at the time of pneumonitis
Cytokeratin 19 Ag	IL 17 A	Ca125
CA242		Angiotensin II type 1 receptor
CCL3		CMT ext
		SOX2
		IL12
		CEA
		Enolase
		SSCA
		Beta Glucuronidase
		CMV G and M

Left panel (yellow) is a list of elevated Ig G auto reactive antibodies only at baseline in patients who experienced pneumonitis during ICI therapy compared with baseline samples of patients with no irAE during therapy. Middle panel (blue) is a list of elevated Ig G auto reactive antibodies not only at baseline (in patients with pneumonitis compared with baseline samples of patients with no irAE) but also elevated during pneumonitis event (when compared with patients' samples who received 12 weeks of ICI and had no irAE). Right panel (green) is a list of elevated Ig G auto reactive antibodies only at the time of pneumonitis event compared with patients' samples who received 12 weeks of ICI and had no irAE)

Supplementary table 5. List of IgM Auto reactive antibodies elevated at baseline and at the time of pneumonitis event

Elevated IgM auto reactive antibodies at baseline	Elevated IgM auto reactive antibodies both at baseline and pneumonitis	Elevated IgM auto reactive antibodies at the time of pneumonitis
Angiotensin II type 1 receptor	ACHRG (cholinergic receptor nicotinic gamma subunit)	Alpha-fodrin
Apolipoprotein B/E	Alpha fetoprotein	BAFF
BRCA1 and BRCA2	Aldolase (muscle)	Beta-glucuronidase
CA19-9	Aldolase-C	CA27-29
CCL5	Alpha 2 macroglobulin-like 1	CA50
CDK2	CA125	CA242
DDX53	CA72-4	Carbonic anhydrase 6
Endothelial cell extract	CCL3	CD255
Glutamate decarboxylase-65/GAD2	CMV (G and M)	Carcinoembryonic antigen
HPV E6	CMV EXT	dsDNA
HSV 1 and HSV 2 antigen	CMV grade III antigen	Enolase
IL-17A	CXCL10	ErbB3 and ErbB4
NY-ESO1	Cytokeratin 19	FUCA 1
Outer membrane protein-C (E.coli) O	EGF receptor	Galactocerebroside
Prostate-specific antigen	ErbB2	GBM
rhHSPG2	Ferritin	GBU-4-5/TDRD-12
RSV antigen	HPV E7 (16+18)	HuD/ELAVL4
Rubeola antigen	IFN alpha and beta	IL-6
Troponin I	IFN-gamma	Lactoferrin
Vascular endothelial growth factor-16	IL-1 alfa and beta	MAGE A4
	IL-2	PKM2
	IL-8	PSMA
	IL-12	rhHSPG2
	LC1	Ro-SSA
	Lysosomal-associated protein 2	Rotavirus SA-11
	MAGE A3	RSV antigen
	MBP	SCCA
	Matrix metalloproteinase-2	SOX-2
	Myelin-associated glycoprotein	VZV antigen
	NSE	
	OX40L	
	Peptidyl arginine deiminases 1-4	
	PD-1	
	Prostatic acid phosphatase	
	Prostate-specific membrane antigen	
	Ribosomal phosphatase protein P0 P1 P2	
	Rubella virus grades III and IV	
	Tetanus toxoid	
	Thrombopoietin	
	Thyroglobulin	
	Tissue transglutaminase	
	Toxoplasma	

Left panel (yellow) is a list of elevated Ig M auto reactive antibodies only at baseline in patients who experienced pneumonitis during ICI therapy compared with baseline samples of patients with no irAE during therapy. Middle panel (blue) is a list of elevated Ig M auto reactive antibodies not only at baseline (in patients with pneumonitis compared with baseline samples of patients with no irAE) but also elevated during pneumonitis event (when compared with patients' samples who received 12 weeks of ICI and had no irAE). Right panel (green) is a list of elevated Ig M auto reactive antibodies only at the time of pneumonitis event compared with patients' samples who received 12 weeks of ICI and had no irAE)

6) FALSE DISCOVERY AND P VALUES FOR AUTO REACTIVE ANTIBODIES AT BASELINE

Baseline auto reactive antibodies during pneumonitis, dermatitis, hepatitis and diarrhea/colitis compared the auto reactive antibody panel with non irAE group (baseline samples of patients who did not have any immune related adverse events during at least 6 months of follow up (control)). Each organ specific toxicity analyzed separately.

Only auto reactive antibodies with a of p-value less than 0.05 has been summarized in below tables. False discovery rate and Fold change also provided in the table 6.1.A. and 6.1.B. for identified antibodies.

Supplementary tables 6.1.A. and 6.1.B. Supplementary tables for PNEUMONITIS, baseline auto reactive antibody panel comparison for pneumonitis vs. non-irAE group, Ig G and Ig M:

Supplementary table 6. 1.A) Ig G

Marker	Pvalue	FDR	FC
Cytokeratin 19 Ag	0.004843	0.619958	3.903535
IL-17A	0.019349	0.98776	1.805318
CA242	0.034922	0.98776	2.008778
CCL3	0.042168	0.98776	2.364204

P value significant if ≤0.05, FDR: False discovery rate, FC: Fold change

Supplementary table 6.1.B) IgM

Marker	Pvalue	FDR	FC
OmpC (E coli outer membrane porin)	0.00047	0.057215	4.351483
HSV-1 and HSV-2 antigen	0.002661	0.057215	4.254834

IL-2	0.002973	0.057215	3.452312
Alpha-2-macroglobulin-like 1	0.003656	0.057215	2.939609
IL-1 alpha and beta	0.003969	0.057215	3.478609
IFN-gamma	0.004024	0.057215	2.913485
BRCA1 and BRCA2	0.004426	0.057215	3.895652
Ox40L	0.005105	0.057215	2.644983
IFN alpha and beta	0.005569	0.057215	2.412754
LC1	0.005701	0.057215	2.926783
MMP-2	0.005891	0.057215	3.396391
Toxoplasma	0.00614	0.057215	2.189032
MBP	0.006152	0.057215	2.516902
MAG	0.006543	0.057215	4.269352
Ferritin	0.007261	0.057215	2.664499
rhHSPG2	0.007684	0.057215	2.917175
VEGF-165	0.007886	0.057215	3.131167
HPV E7 (16+18)	0.009332	0.057215	3.919762
IL-8	0.00948	0.057215	2.616495
Tissue Transglutaminase (TTG)	0.009831	0.057215	2.139732
NY-ESO-1	0.009836	0.057215	2.553587
MAGEA3	0.010134	0.057215	2.40144
CA72-4	0.010281	0.057215	3.286973
Prostate Specific Antigen (PSA)	0.012214	0.062108	2.532473
Rubella virus grade III and IV antigen	0.012827	0.062108	2.028573
CCL3	0.012947	0.062108	1.986482
Cytokeratin 19 Ag	0.013101	0.062108	2.366583
Troponin I	0.01508	0.068939	2.03792
PSMA	0.017032	0.070354	1.845458
ErbB2	0.017147	0.070354	2.796617
EGF Receptor	0.018002	0.070354	2.319584
IL-12 (p70)	0.018236	0.070354	2.883134
CDK2	0.018948	0.070354	2.177329
CMV EXT	0.019539	0.070354	2.692344
Aldolase (muscle)	0.019889	0.070354	2.021209
Ribo Phospha Protein P0 P1 P2	0.02038	0.070354	2.157969
CCL5	0.020914	0.070354	2.848887
Angiotensin II type 1 Receptor	0.021774	0.070354	2.217693
Rubeola antigen	0.021971	0.070354	3.392269
RSV antigen	0.021985	0.070354	2.297203
Cholinergic Receptor Muscarinic 3 (CHRM3)	0.023085	0.072071	2.586711
CMV (G and M)	0.0276	0.084114	2.94248
NSE	0.030239	0.085841	2.446971
HPV E6 (16)	0.030372	0.085841	1.876991

Peptidyl Arginine Deiminases 1-4	0.030682	0.085841	2.32694
CMV GRADE III ANTIGEN	0.030849	0.085841	1.911743
IL-17A	0.032734	0.087593	1.892197
DDX53	0.033702	0.087593	1.78545
AFP	0.033925	0.087593	3.353368
PD-1	0.034602	0.087593	2.735907
Thrombopoietin (TPO)	0.0349	0.087593	3.828593
Apolipoprotein B/E	0.035692	0.087857	2.196748
Prostatic Acid Phosphatase	0.039089	0.091653	1.910915
ALDOC	0.039392	0.091653	1.898625
Glutamate decarboxylase-65/GAD2	0.039722	0.091653	2.214697
Endothelial Cell Extract	0.040098	0.091653	1.835706
Thyroglobulin	0.043333	0.097309	2.457709
CA125	0.044222	0.097593	9.390235
Tetanus toxoid	0.046764	0.100224	1.91369
Lysosomal Associated Protein 2 (LAMP-2)	0.04698	0.100224	2.054589
CXCL10	0.048231	0.101205	2.315798
CA19-9	0.049492	0.102176	3.944197

P value significant if ≤0.05, FDR: False discovery rate, FC: Fold change

Supplementary tables 6.2.A. and 6.2.B. Supplementary tables for DERMATITIS, baseline auto reactive antibody panel comparison for dermatitis vs. non-irAE group, Ig G and Ig M:

Supplementary table 6.2.A. IgG

Marker	Pvalue	FDR	FC
dsDNA	0.038594	0.997145	1.868919

P value significant if ≤0.05, FDR: False discovery rate, FC: Fold change

Supplementary table 6.2.B. IgM

Marker	Pvalue	FDR	FC
IFN alpha and beta	0.018835	0.456247	1.600547
IL-2	0.024212	0.456247	1.833213
Calreticulin	0.041953	0.456247	1.455002
Troponin I-T-C ternary complex mixture	0.043846	0.456247	2.005458

P value significant if ≤0.05, FDR: False discovery rate, FC: Fold change

Supplementary tables 6.3.A. and 6.3.B. DIARRHEA/COLITIS, baseline auto reactive antibody panel comparison for diarrhea/colitis vs. non-irAE group, Ig G and Ig M:

Supplementary tables 6.3.A. Ig G

None

Supplementary tables 6.3.B. IgM

Marker	P value	FDR	FC
Hepatitis A antigen	0.008004	0.261828	2.325275
Thrombopoietin (TPO)	0.008191	0.261828	2.964102
VEGF-165	0.012916	0.261828	2.001886
ACHRG	0.016753	0.261828	2.007051
Rubeola antigen	0.017204	0.261828	2.257629
DDX53	0.021716	0.261828	1.597522
RSVP antigen	0.027321	0.261828	1.957825
genomic DNA	0.027475	0.261828	2.249969
NSE	0.028795	0.261828	1.992499
NY-ESO-1	0.029413	0.261828	1.595
BAFF	0.033133	0.261828	1.701828
Tetanus toxoid	0.041259	0.261828	1.684679
dsDNA	0.041653	0.261828	2.510088
Enolase	0.0439	0.261828	1.64521
Toxoplasma	0.047064	0.261828	1.572502

P value significant if ≤0.05, FDR: False discovery rate, FC: Fold change

Supplementary tables 6.4.A. and 6.4.B. HEPATITIS, baseline auto reactive antibody panel comparison for hepatitis vs. non-irAE group, Ig G and Ig M:

Supplementary table 6.4.A. Ig G

None

Supplementary table 6.4.B. Ig M

Marker	Pvalue	FDR	FC
CDK2	0.007867	0.351345	2.86715
IL-2	0.017962	0.351345	2.927375
Troponin I-T-C ternary complex mixture	0.018433	0.351345	3.915224
HSV-1 and HSV-2 antigen	0.029983	0.351345	3.446584
Rubeola antigen	0.033589	0.351345	3.499312

Saccharomyces Cerevisiae	0.03516	0.351345	2.460669
PD-1	0.041641	0.351345	3.083865
CA72-4	0.048822	0.351345	2.621628
Enolase	0.049918	0.351345	2.136144

P value significant if ≤0.05, FDR: False discovery rate, FC: Fold change

7) FALSE DISCOVERY AND P VALUES FOR AUTO REACTIVE ANTIBODIES AT TOXICITY

Auto reactive antibody panel at pneumonitis, dermatitis, hepatitis and diarrhea/colitis compared to 12-week control blood from patients who did not have any immune related adverse events. Each organ specific toxicity analyzed separately.

Only auto reactive antibodies with a of p-value less than 0.05 have been summarized in below tables. IRAE event

Supplementary tables 7.1.A. and 7.1.B. Antibody panel comparison for pneumonitis, at the time of toxicity event vs. 12-week control blood from non-irAE group

Supplementary table 7.1.A. IgG

Marker	Pvalue	FDR	FC
CA125	0.001817	0.033222	2.946284
CMV EXT	0.011699	0.166389	4.66718
Angiotensin II type 1 Receptor	0.01311	0.167805	2.581111
SOX2	0.022562	0.262538	3.723643
CEA	0.039089	0.324464	6.352035
Beta-glucuronidase	0.042953	0.324464	1.991825
IL-12 (p70)	0.04428	0.324464	1.635602
Enolase	0.046512	0.324464	2.305693
SCCA	0.047879	0.324464	1.53579
CMV (G and M)	0.048064	0.324464	3.838923
IL-17A	0.048163	0.324464	1.55723

P value significant if ≤0.05, FDR: False discovery rate, FC: Fold change

Supplementary table 7.1.B. Ig M

Marker	Pvalue	FDR	FC
ACHRG	0.000185	0.012082	2.900841
NSE	0.000416	0.012082	3.7469
BAFF	0.000416	0.012082	2.79236
Ox40L	0.000474	0.012082	2.872867
SCCA	0.000723	0.012082	2.797048
IL-6	0.000738	0.012082	2.654305
LC1	0.000872	0.012082	2.532841
Beta-glucuronidase	0.000936	0.012082	2.79634
Cytokeratin 19 Ag	0.000995	0.012082	3.49091
Toxoplasma	0.00111	0.012082	2.406376
CA72-4	0.001457	0.012082	3.831821
Prostatic Acid Phosphatase	0.001515	0.012082	2.464917
IL-12 (p70)	0.001519	0.012082	2.985453
CA27-29	0.001644	0.012082	2.664336
VZV antigen	0.001656	0.012082	2.460694
RSV antigen	0.001699	0.012082	3.239714
CMV EXT	0.002045	0.013321	3.127093
CXCL10	0.002179	0.013321	2.861287
CA242	0.002309	0.013321	2.9559
IFN-gamma	0.002389	0.013321	2.588334
Tetanus toxoid	0.002394	0.013321	2.334613
IL-2	0.002498	0.013321	3.328424
IL-8	0.002775	0.014206	2.74355
SOX2	0.003021	0.014872	8.122063
GBU4-5/TDRD-12	0.003723	0.017018	2.159324
MBP	0.004138	0.018265	2.35889
dsDNA	0.004366	0.018627	4.146749
Tissue Transglutaminase (TTG)	0.004652	0.018851	2.036144
MAGEA3	0.004713	0.018851	2.308581
PSMA	0.004903	0.019016	1.98888
Peptidyl Arginine Deiminases 1-4	0.005091	0.019166	2.789099
Ro-SSA (52 + 60)	0.00526	0.019235	2.40439
CA50	0.005544	0.019288	2.431079
Rotavirus SA-11	0.005819	0.019288	2.311478
Lysosomal Associated Protein 2 (LAMP-2)	0.006008	0.019288	2.142733
rhHSPG2	0.006013	0.019288	2.354179
EGF Receptor	0.006095	0.019288	2.67919
CMV (G and M)	0.006178	0.019288	3.626738
Ferritin	0.006959	0.021207	2.379112

MAGEA4	0.00957	0.027632	2.315599
AFP	0.0097	0.027632	4.676667
Galactocerebroside	0.009715	0.027632	2.5634
Ribo Phospha Protein P0 P1 P2	0.010254	0.028331	2.244509
CEA	0.010403	0.028331	3.993001
Lactoferrin	0.013171	0.033981	2.164509
ErbB2	0.013274	0.033981	2.599646
IL-1 alpha and beta	0.01398	0.035087	2.540818
FUCA1	0.014676	0.036125	2.487159
ALDOC	0.01588	0.038351	2.36874
ErbB3 and ErbB4	0.016414	0.038394	2.236071
TWEAK (CD255)	0.016497	0.038394	2.415317
Thyroglobulin	0.017033	0.038933	2.464532
MAG	0.020889	0.046909	2.727135
Alpha-fodrin	0.022722	0.050146	1.965483
Rubella virus grade III and IV antigen	0.023812	0.051659	1.806415
PD-1	0.025594	0.054601	2.460554
CMV GRADE III ANTIGEN	0.0287	0.059171	2.004832
Enolase	0.028792	0.059171	1.744474
MMP-2	0.029123	0.059171	2.446438
Thrombopoietin (TPO)	0.032751	0.065501	2.785395
Alpha-2-macroglobulin-like 1	0.033373	0.06572	2.103491
Cholinergic Receptor Muscarinic 3 (CHRM3)	0.035743	0.068909	2.125234
Prostate Specific Membrane Antigen	0.03607	0.068909	1.696648
HuD/ELAVL4	0.03662	0.068932	1.915403
CCL3	0.037993	0.070479	1.665462
Carbonic Anhydrase 6 (CA6)	0.039482	0.072196	1.797295
PKM2	0.042244	0.076158	1.863357
Aldolase (muscle)	0.045024	0.080042	1.732216
CA125	0.046933	0.082294	9.874557
GBM	0.047861	0.082786	1.753366
HPV E7 (16+18)	0.048712	0.082945	2.259987
IFN alpha and beta	0.049249	0.082945	1.778036

P value significant if ≤0.05, FDR: False discovery rate, FC: Fold change

Supplementary tables 7.2.A. and 7.2.B. Antibody panel comparison for dermatitis, at the time of toxicity event vs. 12 week control blood from non-irAE group

Supplementary table 7.2.A. Ig G

None

Supplementary table 7.2.B. IgM

Marker	Pvalue	FDR	FC
Cytokeratin 19 Ag	0.016652	0.856594	2.004623

P value significant if ≤0.05, FDR: False discovery rate, FC: Fold change

Supplementary tables 7.3.A. and 7.3.B. Antibody panel comparison for diarrhea/colitis, at the time of toxicity event vs. 12-week control blood from non-irAE group

Supplementary table 7.3.A.

Ig G none

Supplementary table 7.3.B.

Ig M none

Supplementary tables 7.4.A. and 7.4.B. Antibody panel comparison for hepatitis, at the time of toxicity event vs. 12-week control blood from non-irAE group

Supplementary table 7.4.A. IgG

Marker	Pvalue	FDR	FC
Thyroglobulin	0.008275	0.433068	3.261201

P value significant if ≤0.05, FDR: False discovery rate, FC: Fold change

Supplementary table 7.4.B. IgM

None

8) LONGITUDINAL CHANGES FOR AUTO REACTIVE ANTIBODIES IN TOXICITY GROUP WITH FALSE DISCOVERY AND P VALUES

Longitudinal blood samples compared at baseline and at the time of toxicity for pneumonitis, dermatitis, hepatitis and diarrhea/colitis. Each organ specific toxicity analyzed separately. Only auto reactive antibodies with a of p-value less than 0.05 has been summarized in below tables. IRAE event

Supplementary tables 8.1.A. and 8.1.B. Antibody panel comparison at baseline and at the time of toxicity for pneumonitis

Supplementary table 8.1.A. IgG

Marker	FC	Pvalue	FDR
CA15-3	1.391581	0.015709	0.67023
PD-1	1.821185	0.041548	0.714324
CMV GRADE III ANTIGEN	2.076585	0.048752	0.714324

P value significant if ≤0.05, FDR: False discovery rate, FC: Fold change

Supplementary table 8.1.B. IgM

Marker	FC	Pvalue	FDR
ACHRG	2.440366	0.030185	0.482962
Calmodulin (brain)	1.761404	0.04212	0.599041

P value significant if ≤0.05, FDR: False discovery rate, FC: Fold change

Supplementary tables 8.2.A. and 8.2.B. Antibody panel comparison at baseline and at the time of toxicity for dermatitis

Supplementary table 8.2.A. IgG

Marker	FC	Pvalue	FDR
PD-1	4.785258	0.000389	0.04985
CTLA4	1.355146	0.011895	0.453018
Cytokeratin 19 Ag	**1.820377**	0.016868	0.453018
CA242	1.405426	0.017328	0.453018
Thyroglobulin	1.487418	0.017696	0.453018
CA50	1.188069	0.03063	0.653435
PD-L1	1.377312	0.042776	0.782187

P value significant if ≤0.05, FDR: False discovery rate, FC: Fold change

Supplementary table 8.2.B. IgM

Marker	FC	Pvalue	FDR
Cytokeratin 19 Ag	2.278222	0.014622	0.935838

P value significant if ≤0.05, FDR: False discovery rate, FC: Fold change

Supplementary tables 8.3.A. and 8.3.B. Antibody panel comparison at baseline and at the time of toxicity for diarrhea/colitis

Supplementary table 8.3.A. IgG

Marker	FC	Pvalue	FDR
PD-1	3.658693	1.03E-05	0.001318

P value significant if ≤0.05, FDR: False discovery rate, FC: Fold change

Supplementary table 8.3.B. Ig M

None

Supplementary tables 8.4.A. and 8.4.B. Antibody panel comparison at baseline and at the time of toxicity for hepatitis

Supplementary table 8.4.A. Ig G

Marker	FC	Pvalue	FDR
TGF-beta1	1.436922	0.003074	0.393525
Lactoferrin	1.251526	0.026332	0.84264
Thyroglobulin	6.135744	0.036159	0.84264

P value significant if ≤0.05, FDR: False discovery rate, FC: Fold change

Supplementary table 8.4.B. Ig M

None

Supplementary tables 8.5.A. and 8.5.B. Antibody panel comparison at baseline and at the end of induction for pneumonitis

Supplementary table 8.5.A IgG

Marker	FC	Pvalue	FDR
PD-1	7.851721131	0.000983718	0.125915845
IFN-r	-1.29097163	0.005932673	0.379691067
CA15-3	1.226290415	0.011162789	0.476278985

Supplementary table 8.5.B. IgM

None

Supplementary tables 8.6.A. and 8.6.B. Antibody panel comparison at baseline and at the end of induction for dermatitis

Supplementary table 8.6.A IgG

Marker	FC	Pvalue	FDR
PD-1	8.091653015	1.95E-05	0.002492495
CA242	1.318440872	0.008534644	0.449927412
CTLA4	1.271357611	0.010545174	0.449927412
Thyroglobulin	1.564046903	0.041630237	0.638077109

P value significant if ≤0.05, FDR: False discovery rate, FC: Fold change

Supplementary table 8.6.B. IgM

Marker	FC	Pvalue	FDR
Cytokeratin 19 Ag	2.253686034	0.003547101	0.395050598
CA242	1.623225592	0.006172666	0.395050598
HuD/ELAVL4	1.26791986	0.030035606	0.605413722
BAFF	1.400794242	0.03141111	0.605413722
CDK2	1.349071943	0.03329702	0.605413722
P53	1.304894672	0.047240058	0.605413722
Angiotensin II type 1 Receptor	1.627571224	0.049174049	0.605413722

P value significant if ≤0.05, FDR: False discovery rate, FC: Fold change

Supplementary tables 8.7.A. and 8.7.B. Antibody panel comparison at baseline and at the end of induction for diarrhea/colitis

Supplementary table 8.7.A IgG

Marker	FC	Pvalue	FDR
PD-1	6.467974045	6.38E-06	0.000817235
Angiotensin II type 1 Receptor	1.203557096	0.027484903	0.634778899
Cytokeratin 19 Ag	1.586422923	0.028519128	0.634778899
CTLA4	1.220990696	0.033394471	0.634778899
CA242	1.375355232	0.034714471	0.634778899
RSV antigen	1.132981458	0.043533362	0.69227931

P value significant if ≤0.05, FDR: False discovery rate, FC: Fold change

Supplementary table 8.7.B. IgM

None

Supplementary tables 8.8.A. and 8.8.B. Antibody panel comparison at baseline and at the end of induction for hepatitis

Supplementary table 8.8.A IgG

Marker	FC	Pvalue	FDR
Prostatic Acid Phosphatase	1.160323632	0.005435085	0.373331431
TGF-beta1	1.720855941	0.005833304	0.373331431
Thyroglobulin	8.791603298	0.01042671	0.435367164
PSMA	1.182967405	0.015069681	0.435367164
PD-1	4.441416902	0.01700653	0.435367164
Beta-glucuronidase	1.318528755	0.026115771	0.477545522
GBU4-5/TDRD-12	1.157427577	0.045394	0.64560356

P value significant if ≤0.05, FDR: False discovery rate, FC: Fold change

Supplementary table 8.8.B. IgM

None

9) LONGITUDINAL CHANGES FOR AUTO REACTIVE ANTIBODIES IN NO TOXICITY GROUP WITH FALSE DISCOVERY AND P VALUES

Longitudinal blood samples compared at baseline and at 12 weeks in patients who did not have any immune related adverse events during at least 6 months of follow up (control). Each organ specific toxicity analyzed separately.

Only auto reactive antibodies with a of p-value less than 0.05 has been summarized in below tables. IRAE event

Supplementary tables 9.A. and 9.B. Antibody panel comparison at baseline and in 12 weeks in no irAE group

Supplementary table 9.1.A. Ig G

Marker	FC	Pvalue	FDR
PD-1	5.820913	4.71E-12	6.03E-10
Prostatic Acid Phosphatase	1.177405	0.000203	0.010746
MAGEA4	1.166348	0.000252	0.010746
MAG	1.207892	0.000529	0.016935
CA50	1.183669	0.00133	0.034041
TWEAK (CD255)	1.200066	0.002287	0.040937
Lactoferrin	1.24327	0.002817	0.040937
CA15-3	1.180149	0.002911	0.040937
IL-12 (p70)	1.207435	0.002998	0.040937
Rubeola antigen	1.145529	0.003198	0.040937
AFP	1.277094	0.004859	0.056545
Enolase	1.160605	0.006054	0.060466
TNF-alpha and beta	1.126731	0.006411	0.060466
GBM	1.159052	0.006613	0.060466
Thyroglobulin	1.338449	0.009626	0.082143
BRCA1 and BRCA2	1.519862	0.010919	0.085492
rhHSPG2	1.294204	0.011558	0.085492
dsDNA	1.186232	0.012022	0.085492
SCCA	1.138406	0.014085	0.085529
ACHRG	1.158607	0.014518	0.085529
CTLA4	1.266339	0.014662	0.085529
HPV E7 (16+18)	1.085288	0.014825	0.085529
EGF Receptor	1.564175	0.015369	0.085529
RSV antigen	1.188584	0.01724	0.085955
IL-2	1.300793	0.017431	0.085955

Alpha-2-macroglobulin-like 1	1.15459	0.017637	0.085955
genomic DNA	1.405329	0.018748	0.085955
LC1	1.140303	0.018803	0.085955
IL-8	1.412874	0.019498	0.08606
Catalase	1.144312	0.022277	0.095048
MBP	1.426453	0.023413	0.095865
MAGEA3	1.169542	0.025365	0.095865
Tissue Transglutaminase (TTG)	1.102844	0.025777	0.095865
Aldolase (muscle)	1.139692	0.027005	0.095865
Rotavirus SA-11	1.111267	0.027645	0.095865
FUCA1	1.128885	0.028095	0.095865
Prostate Specific Antigen (PSA)	1.236224	0.028382	0.095865
IL-1 alpha and beta	1.157112	0.02846	0.095865
Alpha-fodrin	1.130194	0.029342	0.096301
PKM2	1.187719	0.032044	0.10254
NY-ESO-1	1.162953	0.034323	0.107156
Toxoplasma	1.179745	0.037734	0.112485
EBV EBNA1	1.1231	0.038842	0.112485
IFN alpha and beta	1.419886	0.039502	0.112485
Ribo Phospha Protein P0 P1 P2	1.134937	0.039545	0.112485
Ferritin	1.155557	0.040465	0.112598
VZV antigen	1.109393	0.041802	0.113843
Sm/RNP	1.159125	0.043217	0.114807
CA242	1.194378	0.04395	0.114807
CA27-29	1.107259	0.046018	0.117806

P value significant if ≤0.05, FDR: False discovery rate, FC: Fold change

Supplementary table 9.1.B. Ig M

Marker	FC	Pvalue	FDR
TWEAK (CD255)	1.279557	0.000641	0.042997
Enolase	1.320217	0.000672	0.042997
Aldolase (muscle)	1.216801	0.001621	0.047378
IL-12 (p70)	1.425789	0.001853	0.047378
MAGEA3	1.284817	0.001881	0.047378
LC1	1.405077	0.002661	0.047378
anti-Ig control 1:2	1.263456	0.002919	0.047378
PD-1	1.482894	0.002961	0.047378
Endothelial Cell Extract	1.233854	0.00381	0.050206
Ig control 1:8	1.148499	0.004239	0.050206

rhHSPG2	1.494526	0.004746	0.050206
Ig control 1:16	1.181706	0.005003	0.050206
Tissue Transglutaminase (TTG)	1.231777	0.00548	0.050206
HPV E7 (16+18)	1.525081	0.005491	0.050206
ERP29	1.432012	0.006296	0.053728
Rubella virus grade III and IV antigen	1.273733	0.006748	0.053987
CA50	1.151134	0.010611	0.079898
Ig control 1:4	1.110331	0.014435	0.086258
Ox40L	1.255488	0.014833	0.086258
OmpC	1.430454	0.015947	0.086258
Rubeola antigen	1.713792	0.015977	0.086258
Tetanus toxoid	1.231265	0.016208	0.086258
PD-L1	1.464174	0.016536	0.086258
IFN-r	1.360689	0.017086	0.086258
Prostatic Acid Phosphatase	1.215336	0.017932	0.086258
dsDNA	1.500508	0.018461	0.086258
IFN alpha and beta	1.281922	0.018643	0.086258
CMV GRADE III ANTIGEN	1.258457	0.018869	0.086258
anti-Ig control 1:8	1.191849	0.023034	0.095468
Ribo Phospha Protein P0 P1 P2	1.205245	0.024393	0.095468
IL-1 alpha and beta	1.385588	0.024613	0.095468
Carbonic Anhydrase 6 (CA6)	1.24751	0.025149	0.095468
ANXA1	1.857097	0.025175	0.095468
Saccharomyces Cerevisiae	1.276888	0.025915	0.095468
DDX53	1.194252	0.027823	0.095468
Sphingomyelin	1.23021	0.028206	0.095468
VEGF-165	1.292925	0.02823	0.095468
anti-Ig control 1:4	1.278613	0.028959	0.095468
TNF-alpha and beta	1.18627	0.029088	0.095468
Prostate Specific Antigen (PSA)	1.316446	0.03193	0.102177
Alpha-2-macroglobulin-like 1	1.222932	0.033939	0.105163
GBM	1.137006	0.034507	0.105163
CA72-4	1.332648	0.036384	0.108306
GBU4-5/TDRD-12	1.141421	0.038494	0.111983
AFP	1.245907	0.046218	0.131465
CCL3	1.18656	0.047353	0.131765
Ig control 1:2	1.074154	0.049019	0.13265
CMV EXT	1.232647	0.049744	0.13265

P value significant if ≤0.05, FDR: False discovery rate, FC: Fold change

10) IgG ANTIBODY FOR THYROGLOBULIN IN HEPATITIS

Supplemental Figure 2. Box plots for IgG antibody for thyroglobulin

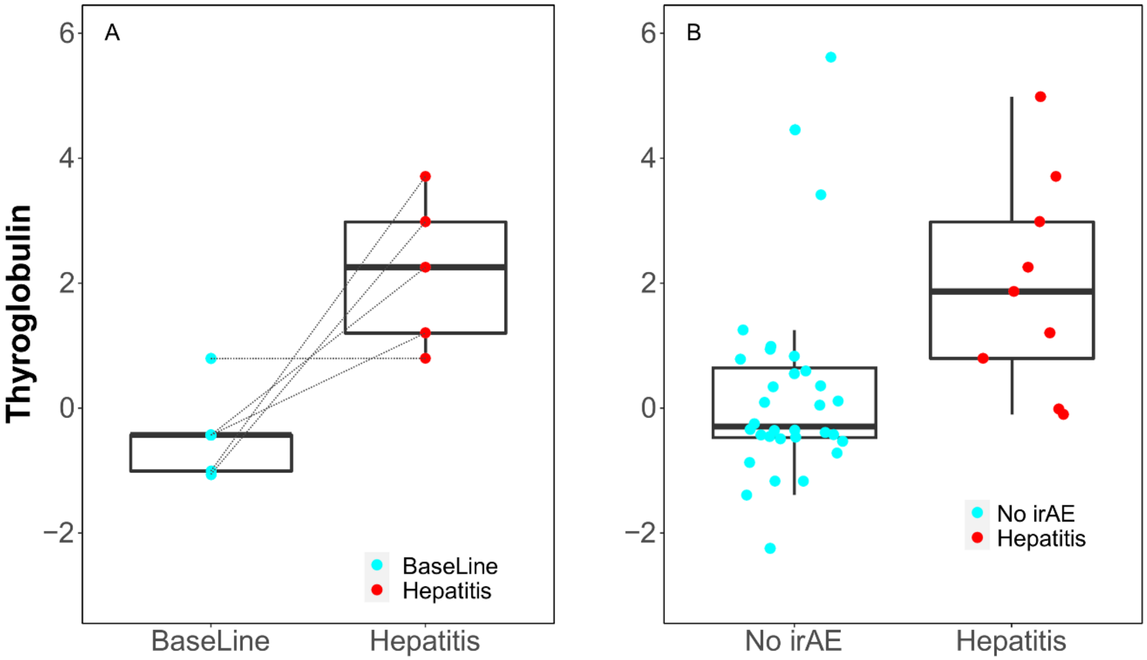

Supplemental figure 2: Box Plots of IgG antibody for Thyroglobulin. (A) Longitudinal serum samples from patients with hepatitis. Baseline: pre-I+N therapy, toxicity: at the time of hepatitis. (B) No irAE: Samples at 12 weeks of I+N therapy from patients with no irAEs. Hepatitis: at the time of grade ≥2 Hepatitis irAE .

11) AUTO REACTIVE ANTIBODY TITERS FOLLOWING STEROID USE:

Supplemental figure 3. Longitudinal Cytokeratin 19 IgM titers in patients with dermatitis

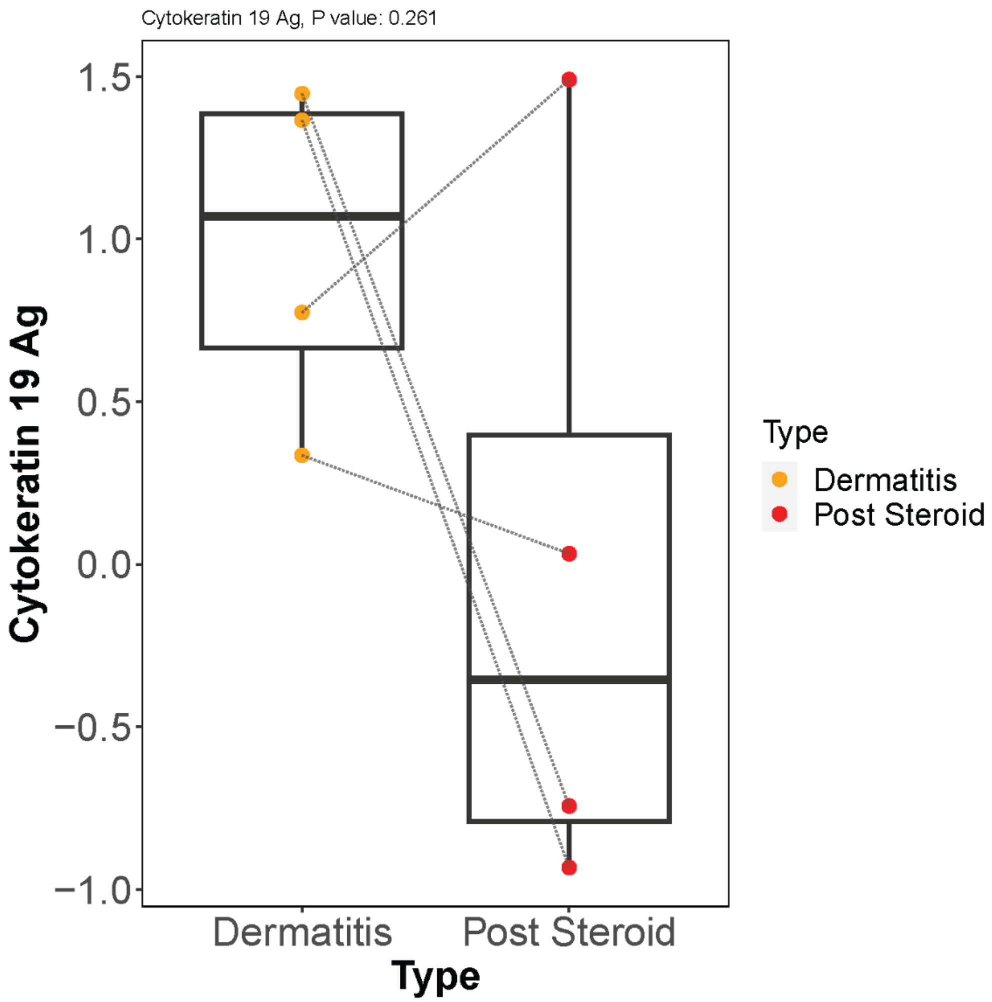

Supplemental figure: Box plots of 4 patients who had G≥2 dermatitis ("dermatitis" samples at the time of toxicity) and treated with steroids ("Post steroid" samples are obtained after the systemic steroid use).

REFERENCES

1. Sims AH, Smethurst GJ, Hey Y, et al. The removal of multiplicative, systematic bias allows integration of breast cancer gene expression datasets - improving meta-analysis and prediction of prognosis. *BMC Med Genomics.* 2008;1:42.
